# Electron Transfer at Quantum Dot–Metal Oxide Interfaces for Solar Energy Conversion

**DOI:** 10.1021/acsnanoscienceau.2c00015

**Published:** 2022-06-22

**Authors:** Marco Ballabio, Enrique Cánovas

**Affiliations:** Instituto Madrileño de Estudios Avanzados en Nanociencia (IMDEA Nanociencia), 28049 Madrid, Spain

**Keywords:** Quantum dots, Metal oxide, Sensitized systems, Electron transfer, Interfacial dynamics, ultrafast spectroscopy, Photovoltaics, Photocatalysis

## Abstract

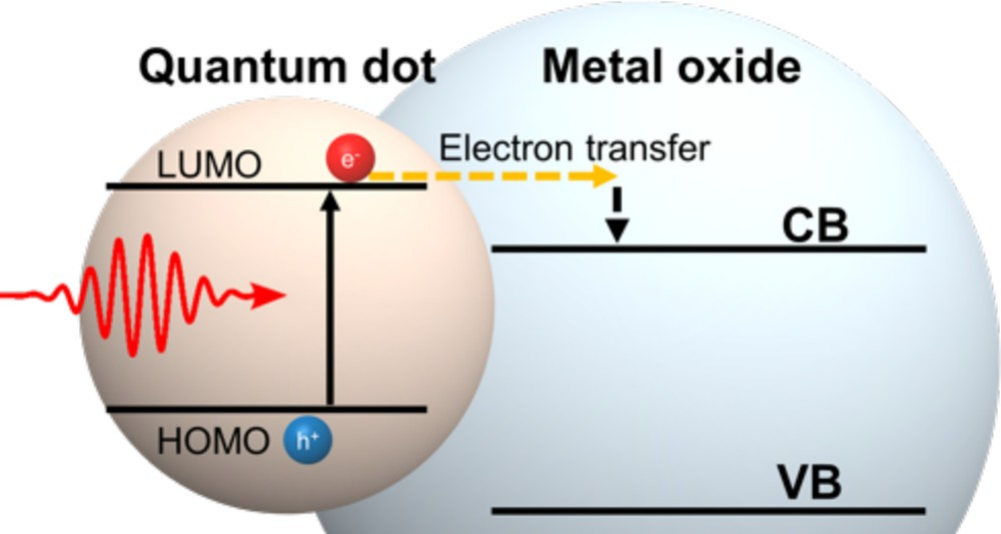

Electron transfer at a donor–acceptor quantum dot–metal oxide interface is a process fundamentally relevant to solar energy conversion architectures as, e.g., sensitized solar cells and solar fuels schemes. As kinetic competition at these technologically relevant interfaces largely determines device performance, this Review surveys several aspects linking electron transfer dynamics and device efficiency; this correlation is done for systems aiming for efficiencies up to and above the ∼33% efficiency limit set by Shockley and Queisser for single gap devices. Furthermore, we critically comment on common pitfalls associated with the interpretation of kinetic data obtained from current methodologies and experimental approaches, and finally, we highlight works that, to our judgment, have contributed to a better understanding of the fundamentals governing electron transfer at quantum dot–metal oxide interfaces.

## 1 Quantum Dot–Metal Oxide (QD–MO) Systems

Metal oxides (MOs) are robust, abundant, and low cost materials exploited in a plethora of applications.^1^ As a drawback, and specifically for solar energy conversion, the optical excitation onset for most MOs is typically prohibitively high for the generation of electron–hole (e-h) pairs through direct absorption of visible light. This obstacle has been circumvented by the sensitization of MOs by impurities,^2^ molecular dyes,^3−6^ and more recently by semiconductor quantum dots (QDs).^7−12^ When a mesoporous MO is employed as an electrode, a large surface-to-volume ratio can be achieved, which allows a high loading of sensitizers to maximize sunlight absorption. Among the multitude of MO materials available, titanium dioxide (TiO_2_) has been the dominant choice.^13^ However, TiO_2_ has few features that can eventually be considered disadvantages for certain applications, e.g., a very modest charge carrier mobility in TiO_2_, a factor that complicates electron charge transport in mesoporous films, and a relatively narrow band gap of 3.2 eV, a gap that enables the absorption of a substantial portion of the UV region of the solar spectrum and can affect eventually the long-term stability in sensitized geometries.^14−16^ To bypass both of these critical issues, materials with better charge transport properties and/or larger band gaps have also been analyzed to a certain extent in the literature, most notably tin dioxide (SnO_2_) and zinc oxide (ZnO).^17,18^

To our knowledge, the first works employing QDs as a sensitizer for a mesoporous metal oxide were published in the early 1990s, where samples consisting InAs, CdSe, CdS, and PbS QDs directly nucleated onto a MO matrix were reported.^19−21^ Later, Zaban et al. functionalized a sintered electrode of 20–25 nm diameter TiO_2_ nanoparticles with colloidal InP quantum dots.^22^ A complete solar cell employing a liquid I^–^/I_3_^–^ or hydroquinone/quinone acetonitrile solution and a Pt counter electrode was assembled and revealed a photocurrent spectrum consistent with the absorption spectrum of the InP dots, a direct proof of efficient electron transfer from the QDs to the MO electrode. All these pioneering works were produced as a natural evolution to the sensitization of MO by molecular dyes, systems studied in depth for the previously developed dye sensitized solar cells (DSSCs).^3−6,23^ Photophysics in dye–MO interfaces have been indeed widely scrutinized, and several good reviews exist on the topic^24−27^ and offer quite relevant information to any reader interested in the topic discussed herein.

**Figure 1:**
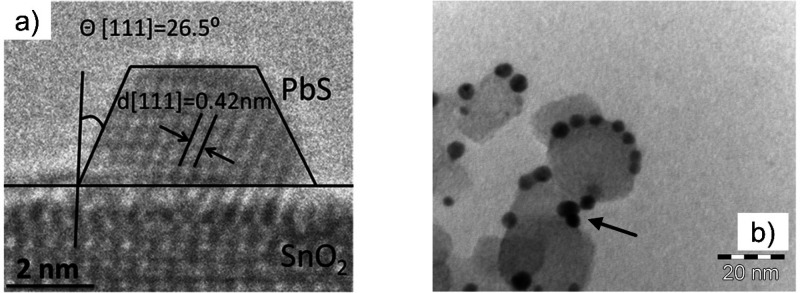
(a) HRTEM of in situ nucleated QDs consisting of SILAR PbS/SnO_2_. Adapted from ref (169). Copyright 2014 American Chemical Society. (b) QD-MPA-TiO_2_ donor–bridge–acceptor from ref (30). Adapted from ref (30). Copyright 2010 American Chemical Society.

In simple terms, the sensitization of MOs by QDs can routinely be achieved by (i) in situ nucleation of QDs directly onto a MO (Figure 1a) or (ii) ex situ preparation of colloidal QDs and subsequent functionalization of the MO (see Figure 1b). The latter can proceed via direct adsorption or be mediated by a bifunctional molecular linker capable of selectively bonding to the oxide electrode and QD.^7−9,28,29^ It is worth noting here that while these geometries are structurally different, they can all be defined energetically as a donor–barrier–acceptor system. Depending on the specific constituents of the QD–MO interface, different elements can play the role of the energy barrier, such as the immediate interface between the dot and oxide (e.g., a monolayer of PbO between PbS and TiO_2_), an air/vacuum gap between the dot and oxide (where the electron needs to be transferred through space), or a molecular linker with a singular chemistry tailored to anchor the QD to the MO surface (where electron is transferred through the bond). As we will discuss in general terms below, the nature of the barrier will fundamentally determine the nature of electron transfer (ET) between the QD and MO, i.e. whether ET occurs via tunneling through a relatively narrow barrier or either hopping through states in the bridging element.

**Figure 2:**
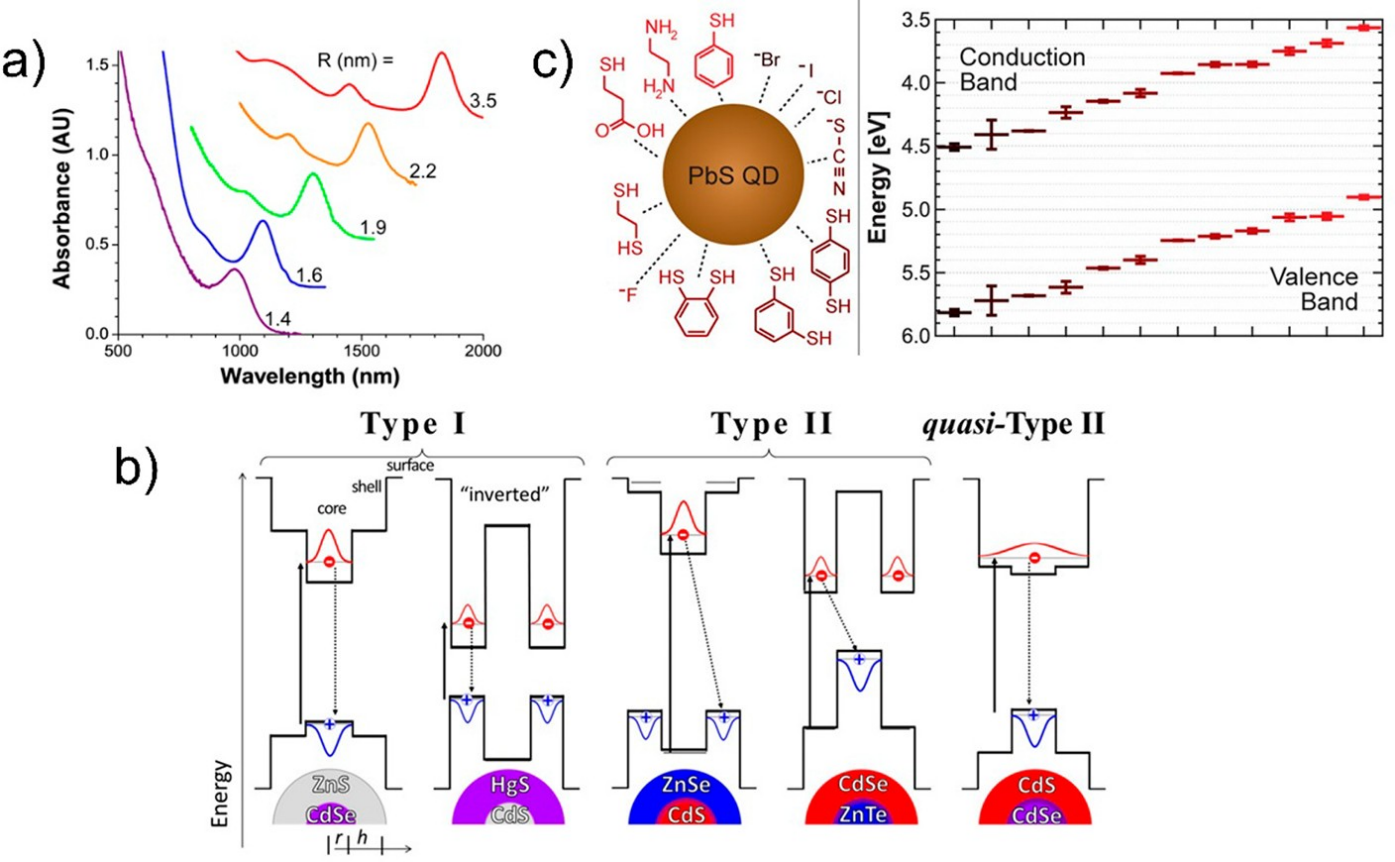
(a) Absorption spectra of PbSe NCs in hexane for different NC sizes (offset for clarity), showing the tuning of the QD gap with particle size. Adapted with permission from ref (93). Copyright 2005 by the American Physical Society. (b) Wave function engineering in QD core–shell heterostructures. Reprinted from ref (53). Copyright 2016 American Chemical Society. (c) Work function tuning. Energy level diagrams of PbS QDs exchanged with the ligands shown around the QD. Reprinted from ref (72). Copyright 2014 American Chemical Society.

Certainly, QDs are very unique building blocks for optoelectronics in general and for energy applications in particular.^7−12,31−33^ QDs are defined by strong absorption cross sections and are very versatile when employed in sensitized MO geometries. This is due to the large degree of tunability that can be achieved in QD physicochemical properties as a function of both nanocrystal morphology and elemental composition. The most prominent optoelectronic feature for QDs linked with morphology is obviously their energy gap tuning by modulating the QD radius (Figure 2a); this phenomenon is described by quantum confinement.^34−37^ Regarding solar energy conversion schemes, QD gap-size modulation is a very appealing feature, which, e.g., lifts the constraints of several low gap bulk materials to be employed in solar energy conversion schemes while possessing an optimal band gap for reaching high efficiency, toward the ∼33% Shockley–Queisser (SQ) limit. QDs have been also proposed as building blocks for developing device architectures with efficiencies beyond the SQ limit, e.g., exploiting gap-size tuning in geometries as the z-scheme in photocatalysis or tandem sensitized solar cells^38−41^ or by exploiting novel and emergent phenomena in QD systems as, e.g., multiple exciton generation or hot electron collection at electrodes (theses aspects are discussed in more depth in section 7).^42−46^

Beyond the size-dependent properties, which enables band gap engineering in the most common “spherically” shaped QD nanostructures, the QD structure can evolve into more complex architectures by following appropriate synthetic routes, producing, e.g., nanorods, 1D and quasi-1D systems, 2D nanoplatelets, tetrapods, etc.^47,48^ These elaborate architectures enable wave function engineering;^49^ this is done by molding the precise spatial localization of electrons and holes wave functions within the nanostructures. A notable example for wave function engineering are core–shell QDs, which can be precisely defined to exhibit a type I or II semiconductor band alignment (Figure 2b). This control of wave function localization has been proven to be very effective in fine-tuning critical aspects such as, e.g., increasing exciton radiative lifetimes, inhibiting Auger recombination, or shifting emission wavelengths.^23,50−52^ Fine tuning these aspects in solar cell architectures is a very appealing and useful feature toward improved performance/functionality/efficiency.

Obviously, the specific chemical composition of a QD fundamentally determines its optoelectronic properties. For example, the range of energies that can be modulated by tuning QD size critically depends on the bulk band gap and the exciton Bohr radius specific to the chosen material.^35−37,53^ As a rule of thumb, having a large Bohr radius will imply that the quantum effects can be observed for a wider range of QD sizes; this is likely one of the reasons why bulk-low-gap lead chalcogenides salts (PbS, PbSe, PbTe) have been widely scrutinized in sensitized architectures, despite their toxicity. Colloidal QDs are typically covered by a corona of organic molecules. Structurally, this ensures the stability of the QD and prevents aggregation and precipitation of the particles when they are in solution.^54,55^ Apart from this, and critically, the molecular capping layer acts also as an electronic passivation layer that reduces or even fully inhibits the detrimental impact of surface recombination centers.^34,56−61^ Furthermore, it is well-known that molecular vibronic states might couple with electron (hole) states in QDs^2,23,62−71^ and molecular entities with a dipole moment can even tune QD work functions (Figure 2c).^72,73^ As such, it is clear that the specificity of the molecular ligands covering the QDs could play an important role when monitoring carrier dynamics in QDs and hence in the interfacial dynamics taking place at QD–MO interfaces.

Apart from the appealing structural and optoelectronic aspects described above, which enable the design of nanostructured systems with tailored properties and hence functionality, both colloidal and in situ nucleated QDs can be produced at room temperature by solution processing. This aspect does have a direct impact in the costs linked with manufacturing, making QDs very appealing as excitonic sensitizers and as building blocks for solar energy conversion schemes.

## 2 Photoconversion Efficiency Limits for Devices Employing QD–MO Interfaces

**Figure 3:**
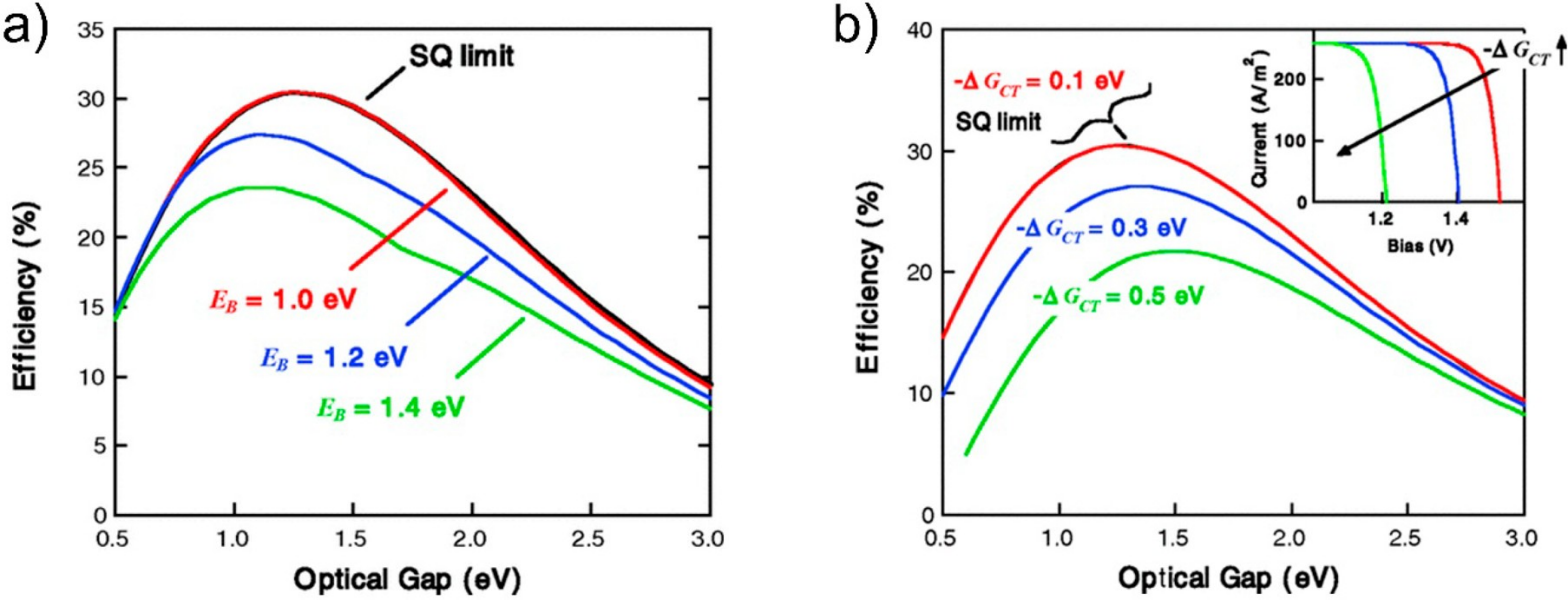
(a) Maximum efficiencies predicted for excitonic solar cells as a function of optical energy gap for several different binding energies (*E*_B_) under 1 sun, assuming the sun is a blackbody with surface temperature *T* = 5778 K. The excitonic limit falls below the SQ limit for *E*_B_ > 1 eV. (b) Maximum efficiency predicted for an ideal donor–acceptor solar cell as a function of exciton optical energy gap and several values of the free energy for charge transfer at the heterojunction, −Δ*G*_CT_. Increased recombination from the lower energy bound-pair states leads to reduced efficiency due to the decrease in open-circuit voltage, as shown in the inset for a cell with *E* = 1.8 eV. Reprinted with permission from ref (76). Copyright 2011 by the American Physical Society.

A solar device based on a single material with a band gap of ∼1.34 eV has an upper threshold efficiency defined by the Shockley–Queisser limit (SQ limit, ∼33% under 1 sun illumination; ∼41% under full solar concentration).^74^ This limit is set by the trade-off for the two major intrinsic loss energy channels occurring in single-gap solar cells: (1) their inability to absorb photons with energy lower than the device band gap and (2) the dissipation as heat (cooling or thermalization) of the excess energy of photogenerated electrons and holes above the band gap. However, the SQ limit is estimated assuming the generation of free, delocalized electrons and holes with unity quantum yield which are collected at selective e and h electrodes without energy loss (i.e., by ohmic contacts). This aspect might differ generally in “excitonic solar cells” like those based on a QD sensitized MO interface.^75^ In this case, the generation of free charges occurs only after the dissociation of the primary photoproduct: a bound exciton in the QD sensitizer. In terms of efficiency, breaking the exciton at the interface requires an energy penalty that is linked with the specific exciton binding energy (*E*_B_ in Figure 3a),^76^ which is inherently a material-dependent property. Furthermore, it is common in “excitonic” QD–MO interfaces to find a large energy offset between the donating QD LUMO and the bottom of the oxide conduction band, which is mainly determined by the equilibration of the chemical potentials of QD and MO constituents at the sensitized interface. This excess energy is commonly referred to as the free energy for charge transfer at the interface and is denoted as Δ*G*. This potential mismatch, which can be considered in a way as a “non-ohmic” contact between donating and accepting states, places an additional constraint on the upper limit efficiency to QD–MO junctions. A large difference between donor and acceptor states is evidenced experimentally in solar cells as a deficit in open circuit voltage (see Figure 3b inset).^76^ From the perspective of kinetics, while reduced Δ*G* values will be preferable toward higher device efficiencies, they are intrinsically linked with slower electron transfer rates from the QD donor to the MO acceptor, as explained in detail in section 6.1. Slow transfer rates can critically compete with other recombination paths within the QDs (both radiative and nonradiative), eventually compromising current collection at the electrodes. This trade-off between voltage and current determined by a kinetic competition at the interface, which is general for all sensitized systems, strongly depends on the specific QD–MO morphology and interfacial chemistry under study; these aspects are discussed in the next section in more detail.

On top of the losses that we have mentioned, devices based on QD–MO interfaces such as QD sensitized solar cells suffer in practice from other extrinsic loss mechanisms; including recombination induced by traps, transmission losses due to poor QD loading, and photostability issues linked with the employed constituents.^8,9,77−79^ In any case, to date, the best performing QD sensitized solar cell reveals a remarkable certified efficiency of 15.2%.^77^ It is worth commenting here that QD sensitized cells are often classified within the general umbrella of “QD solar cells”, where cells employing bulklike QD superlattices hold the record efficiency.^38,80^ We believe this is misleading, as sensitized solar cells employing a dye or a QD as a chromophore operate as excitonic solar cells,^75,76^ while cells employing QD superlattices operate as bulk-like devices relying on the generation of free delocalized charge carriers upon photon absorption.^7,81−85^ While record performing QD sensitized solar cells have reduced efficiencies when compared with those based on QD superlattices, they currently outperform in efficiency their counterpart built around molecular sensitizers (currently delivering cells with about 12% efficiency).^38,80^

Many forecasts indicate that the future of photovoltaics will be linked to the development of more appealing, yet more complex, approaches which demand boosting photoconversion efficiencies for thin film technologies beyond the SQ limit; this is known as third-generation photovoltaics.^38,45,86,87^ These novel approaches aim at overcoming the previously introduced two major intrinsic loss channels occurring in conventional solar cells: (1) their inability to absorb photons with energy less than the device absorption threshold and (2) the waste of photon energy exceeding the band gap (cooling). The routes to surpass the SQ limit can be grouped into three generic categories, namely: (i) multiple energy threshold devices (e.g., multijunction/tandem solar cells and intermediate band solar cells); (ii) the use of excess thermal generation to enhance voltages or carrier collection (e.g., hot carrier solar cells and carrier multiplication); and (iii) the modification of the incident spectrum (e.g., up–down conversion). To date, only solid state multijunction solar cells have shown in practice efficiencies above the SQ limit, reaching figures above 40%,^38^ at costs that regrettably make them not yet competitive against energy production based on fossil fuels.^88^

**Figure 4:**
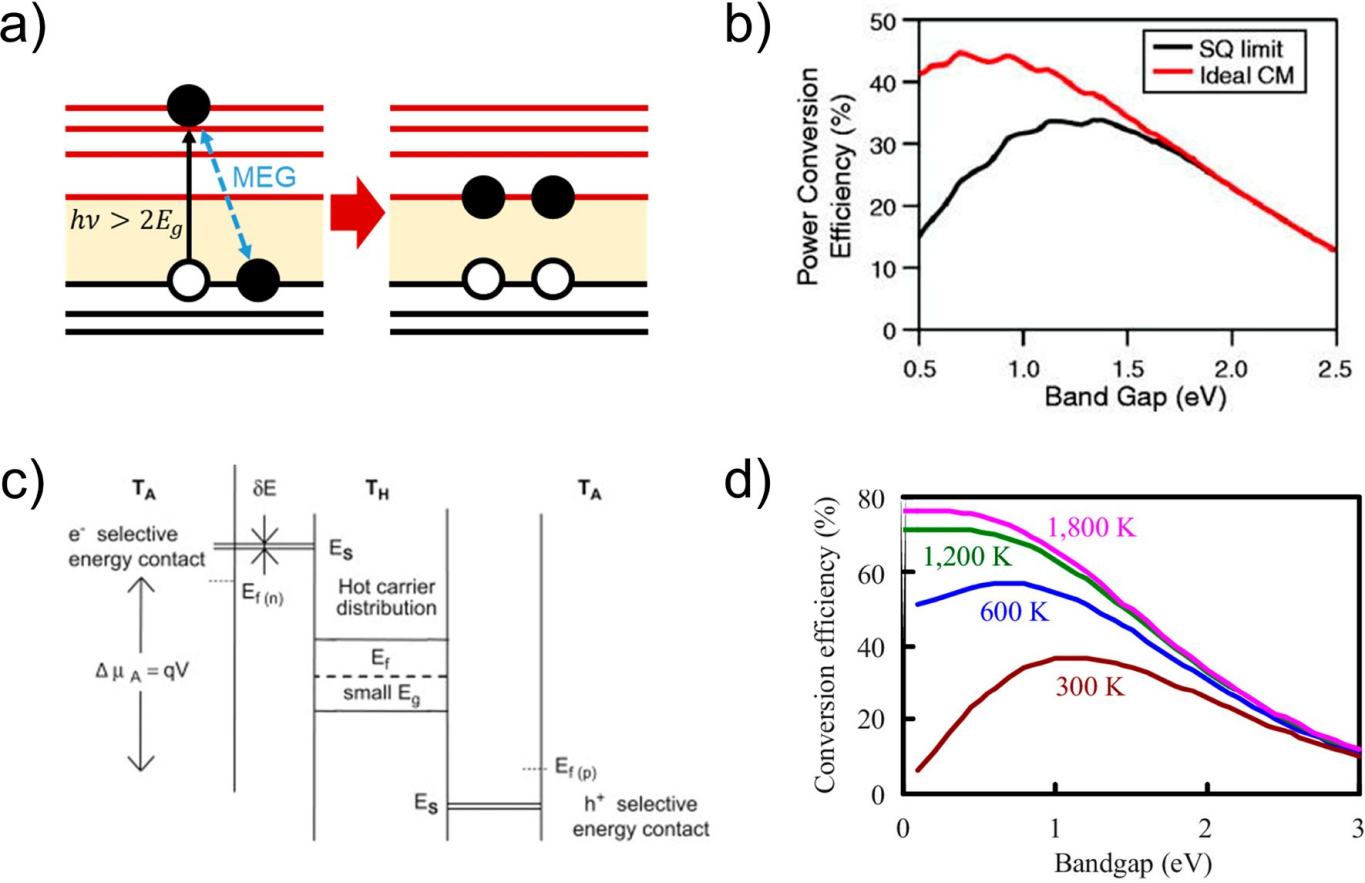
(a) Schematic of multiexciton generation (MEG) after photoexcitation with a highly energetic photon. (b) Theoretical power conversion efficiency without (Shockley–Queisser limit) and with the ideal CM scenario. Adapted from ref (116). Copyright 2018 American Chemical Society. (c) Schematic of the working principle of a hot carrier solar cell. Reprinted with permission from ref (107). Copyright 20089 Elsevier. (d) Theoretical efficiency limit of a hot carrier solar cell, operating at different electron temperatures in the absorber, *T*_H_. Reprinted from ref (108). Copyright 2009 with the permission of AIP Publishing.

Among the third-generation strategies mentioned above, the absorption threshold tunability of semiconductor nanocrystals makes them very attractive for realizing tandem geometries in a cheaper way when compared with their solid state counterparts.^40,41^ Independently of this aspect, likely the most explored third-generation approach employing QDs as active solar absorbers has been carrier multiplication (CM, also known in the literature as multiexciton generation, MEG).^45^ CM refers to the process in which a photogenerated hot charge carrier with an energy of at least *E > 2E*_g_ (where *E*_g_ refers to the HOMO–LUMO gap) promotes another charge carrier across its gap via impact ionization. By doing so, the photon excess energy contained in the hot electron is employed to generate another charge carrier across the gap rather than being wasted as heat. A device using CM could reach theoretically an efficiency above 40% under one sun illumination (see Figure 4).^45,89,90^ Solar cell photocurrent enhancement induced by multiexciton generation from a single absorbed photon was initially proven in bulk silicon devices,^89^ but the improvement in overall device efficiency was marginal. One decade ago, an explosion of work around the CM concept was registered together with the emergence of colloidal QDs, with the expectation of observing high MEG yields compared to bulk absorbers. This push in the field was linked to the expectation of slow hot carrier relaxation in QDs (the so-called phonon-bottleneck effect).^45,91,92^ Over the years, a strong debate followed on whether the phonon bottleneck was indeed operative in QDs^93−100^ and also on whether quantum confinement promotes higher MEG efficiency in nanocrystals with respect to their bulk crystal counterparts.^101−104^ Independently of these aspects, as a proof of concept, QD-based solar cells demonstrating unambiguously a gain in photocurrent in the UV part of the solar spectrum induced by MEG were reported.^42,105^ Several kinetic studies on this topic based on QD–MO systems are highlighted in this Review in section 7.2.

The use of excess thermal generation to enhance voltage inspires the concept of hot carrier solar cells (HCSCs).^43,44^ Although the potential in efficiency gain for HCSCs is among the best envisioned in photovoltaics, HCSCs prototypes are difficult to implement in practice.^106^ In this sense, most of the work reported to date aimed at demonstrating some of the key operation aspects defined by the theory, mostly interrogating avenues to slow down hot carrier cooling and/or boost hot carrier extraction toward a given electrode. Here, however, it is worth noting that while achieving hot electron transfer is a necessary requirement for HCSCs, it alone does not prove the feasibility for the implementation of such a device.^43,44^ Apart from having efficient HET, selective contacts being capable of thermally isolate the absorber need to be engineered, in a way that extraction of hot carriers does not substantially changes the temperature of the electrons in the absorber (Figure 4c).^43,107−109^ This a very demanding and stringent constraint which to the best of our knowledge has not been fulfilled in any of the systems explored so far. Furthermore, while the HCSC concept could theoretically reach an efficiency up to 66% under one sun illumination (Figure 4d),^43,44^ the discretization of energy levels in nanostructures like the QDs might impose another constraint in the implementation of the HCSC concept. The constrain is linked to the definition of a univocal hot temperature for the photogenerated hot carriers in the QD absorber. Electrons populating different electronically isolated states in quantum confined systems are likely not in thermal equilibrium (i.e., each electronic state can be defined by a finite *T*); as such, a low energy electron in the QD LUMO will require the assistance of a second IR photon to gain the required excess energy to reach a higher in energy selective contact.^108,110^ This deviation from theory, imposed by quantum confinement in QD-based systems, makes any approach employing QD–MO interfaces closer to the intermediate band solar cell concept rather than to the HCSCs.^110,111^ In the case a second IR photon is needed to extract an elecron from the absorber toward a selective contact, the upper efficiency limit should be redefined to ∼64%.^112−115^ Independently of these technicalities and practical considerations, within the QD–MO field, many research groups have focused their attention on demonstrating viable extraction of hot electrons populating the QDs toward the MO electrode; highlights of the works on the topic from the perspective of kinetics are made in section 7.1.

## 3 Kinetic Competition at QD–MO Interfaces

The efficiency limits explained in the previous section for a single gap solar cell assume that the two key operation principles of a solar cell are fulfilled with unity quantum yield.^74,76^ The two processes that we refer to are (i) efficient generation of an electron–hole pair upon above-band-gap photon absorption and (ii) the collection of electron and holes in selective contacts. In a QD–MO interface, these two processes take place right at the interface, as such it is widely acknowledged that kinetic competition at the sensitized interface will ultimately determine the efficiency of a solar converter based on these building blocks. In the following, we discuss intrinsic and extrinsic kinetic pathways at QD–MO interfaces; by intrinsic and extrinsic pathways, we refer, respectively, to those that are inherently linked to the nature of the involved constituents (e.g., radiative relaxation in the QD) and those that can be eventually removed by defect engineering (e.g., traps at the surface of the QDs).

**Figure 5:**
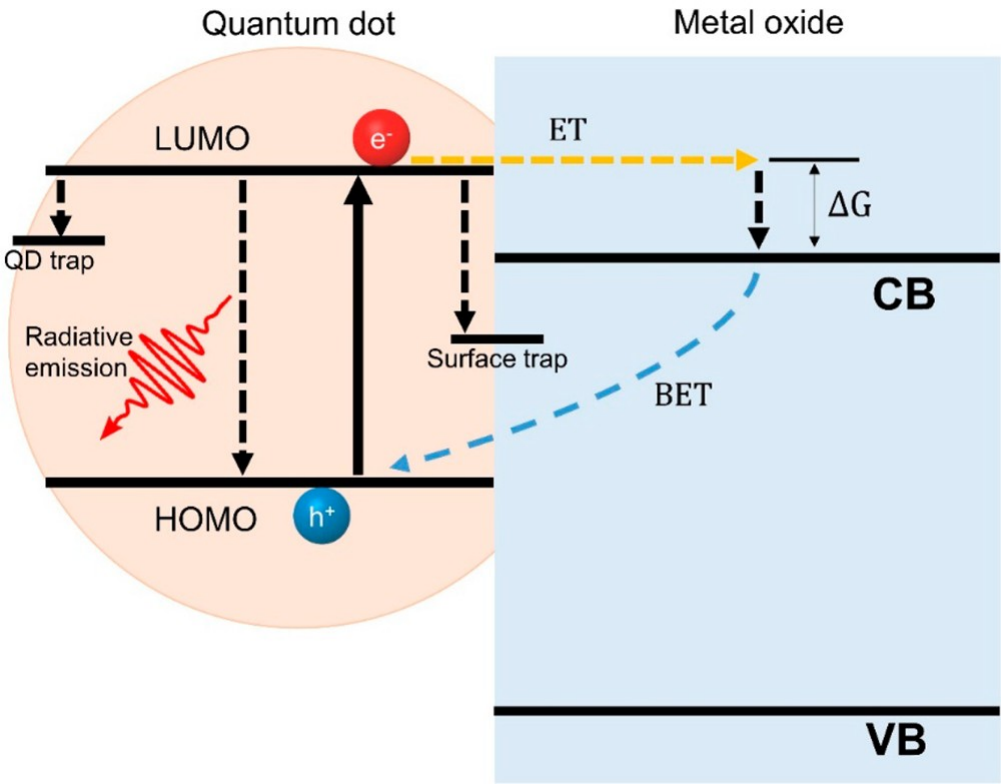
Schematic of the kinetic competition taking place at the QD–MO interface. After the above HOMO–LUMO QD photoexcitation (black solid arrow), electron transfer toward the MO (ET, orange dashed arrow) competes kinetically against radiative recombination and nonradiative trapping at the QD and/or MO surfaces. Once ET takes place, electron transport within the MO will compete kinetically with back electron transfer (BET, blue dashed arrow) toward the QD (and eventually toward a hole populating a hole transporting electrode, not shown here).

In Figure 5, we show a sketch illustrating a QD–MO donor–acceptor (D-A) interface where relevant kinetic pathways are highlighted. After above-HOMO–LUMO-gap photon absorption, an exciton is created within the QD absorber. This exciton can be dissociated at the interface following an electron transfer process toward the MO (denoted as ET, orange dashed arrow in Figure 5); this interfacial exciton dissociation represents a key process for photoconversion, which in QD–MO has to be followed by efficient transport of the photogenerated free electron populating the MO toward an external circuit (in solar cell architectures) or a reaction site at the oxide surface (in solar fuel schemes). As seen in Figure 5, the critically fundamental ET process has to compete kinetically with several relaxation pathways within the QD which are all detrimental for the generation of photocurrent in the MO electrode. In a system lacking any defects, the only competing kinetic channel against ET will be radiative relaxation within the QD for the photogenerated exciton. In this sense, when designing a QD–MO interface, one has to ultimately guarantee that ET competes efficiently with radiative decay, and ideally achieves this employing the lowest Δ*G* offset as briefly described in section 2 (Figure 3b). However, in practice, it is common that several defects populate the samples, as such ET has to compete with extrinsic deactivation pathways linked with charge carrier trapping at the QD or MO surface. Generally speaking the rate constant associated with nonradiative trapping at QD surfaces is few decades faster than radiative relaxation within the QD,^117−119^ as such trapping at the QD surface could be the primary loss of photocurrent in most reports analyzing kinetics at QD–MO interfaces. Trapping at a QD surface is often linked with QD oxidized species or dangling bonds not coordinated with the passivating organic capping ligands.^117−120^ In this sense, trapping at the QD surface is highly dependent on sample nature and chemistry and also on the specific energetics of the traps involved (that in some unusual cases can even have a null effect on photocurrent collection a the MO interface).^121,122^

Following the sketch shown in Figure 5, another extrinsic deactivation channel against ET could be the presence of traps at the MO surface. There is mounting evidence that acceptors at MO surfaces could compete kinetically with ET toward the MO conduction band. For example. several works report ET toward midgap MO surface defects in oxides where ET from the donor to acceptor is forbidden (as, e.g., in ZrO_2_).^30,123,124^ Below the gap, MO band tails are also indicative of the presence of traps in a given MO, traps that can accept electrons as well in the most scrutinized electrodes (TiO_2_, ZnO, and SnO_2_).^125−127^ Also the presence of shallow traps at the oxide surface has been linked to the operating mechanism of charge transport.^128−130^ However, to our understanding, the specific kinetic pathway linked with MO surface traps has not been studied in depth yet. Few reasons might be behind this aspect: extrinsic dopants generating traps at the MO surface are highly dependent on sample history, complicating their identification in a rather complex mesoporous surface. Furthermore, and critically, note that is difficult to resolve them kinetically as their kinetic fingerprint is similar and overlaps with those associated with ET (we describe this in more detail in the following section). In summary, trapping is considered an extrinsic kinetic competition factor that largely depends on the materials of choice, sample preparation, and history. In principle, rational engineering of the surfaces of constituents at the QD–MO interface can largely reduce or even suppress the detrimental effect of these traps by proper passivation.

Provided that ET could eventually compete efficiently with intrinsic and extrinsic deactivation pathways in the QD–MO interface, ET form the QD-LUMO toward the MO conduction band will take place. If, for example, coherent tunneling from donor to acceptor is the main mechanism, ET will occur without energy loss between donor and acceptor and then the transferred electron will have an excess energy Δ*G* when compared to the bottom of the MO-CB. Immediately after the electron populates the oxide CB, this excess energy will be dissipated as heat through the emission of phonons. Once the electron reaches the bottom of the MO-CB, its diffusion-driven transport in the percolated mesoporous MO can be kinetically compromised again by intrinsic and/or extrinsic mechanisms.^128−130^ Trapping at the oxide surface will be an extrinsic mechanism that can be, in principle, engineered out; on the other hand, back electron transfer from the MO-CB to the QD-HOMO (referred to as back electron transfer, BET, in Figure 5) is an intrinsic mechanism that largely depends on the nature of the components and associated interfacial energetics. Obviously, these deactivation mechanisms against electron transport within the MO are also detrimental for the generation of photocurrent in solar energy conversion devices.

Finally, although not shown in Figure 5, a complete solar converter device will require the extraction of the hole from the QD to a selective hole contact; i.e., the hole contained in the QD also needs to be extracted (reduced) by an electrode in solar cells (e.g., an electrolyte or solid state conductor) or directly triggers a chemical reaction at the QD surface in solar fuels (e.g., in water splitting). In a first approximation, hole transfer from the QD to, e.g., a solid state hole selective contact will be defined by the similar kinetic competition against trapping at the QD shell surface and/or back hole transfer from the hole contact toward the dot.^131−137^ On the other hand, it is presumed that the lifetime associated with the “intrinsic” deactivation BET pathway from the MO back to the QD will be largely affected by whether the hole in the QD remains or was efficiently removed from the QD toward a hole selective contact. For the latter case, a new deactivation path linked with back electron transfer from the MO-CB toward the hole transporting material must be considered. In this Review, we primarily focus our discussion on ET from the QD toward the MO for electrodes not containing a hole conductor.^138,139^

## 4 Common Methods for Estimating ET at QD–MO Interfaces

Historically, many powerful techniques have been employed to interrogate charge carrier dynamics at sensitized interfaces such as, e.g., impedance spectroscopy.^140−142^ Among them, spectroscopy approaches based on pump–probe techniques are often the preferred choice when a high time resolution is required. Following these ultrafast spectroscopy schemes, there are two main routes to investigate the ET processes at QD–MO interfaces. The most traditional way is to monitor changes over time after photon absorption in the QD photophysics, e.g., by taking advantage of the fact that an electron transfer event from the donating QD-LUMO toward the MO conduction band leads to a quenching of the QD luminescence of the QD ground state absorption. Alternatively, one can try to trace changes in the optoelectronic properties of the MO acceptor upon arrival of the electron, e.g., the emergence of finite pump induced photoconductivity in the oxide CB following the ET process; this is commonly achieved by following a pump–probe scheme with a UV–vis above-QD-gap pump and a far IR probe or a THz probe (both primarily sensitive to free carriers in the MO-CB).

In this section, we briefly introduce the most common methods that have been employed for characterizing ultrafast ET at QD–MO interfaces, namely, time-resolved photoluminescence (TRPL), transient absorption spectroscopy (TAS), and time-resolved terahertz spectroscopy (TRTS). After presenting the methods, a follow up section will briefly survey the strengths and weaknesses of each technique and discuss common pitfalls that might affect the interpretation of the data arising from all and each of them. However, it is worth stating here that this section does not pretend to be by any means detailed or comprehensive, as many good reviews and books on the different methodologies are currently available.^143−146^

### 4.1 Time-Resolved Photoluminescence

**Figure 6:**
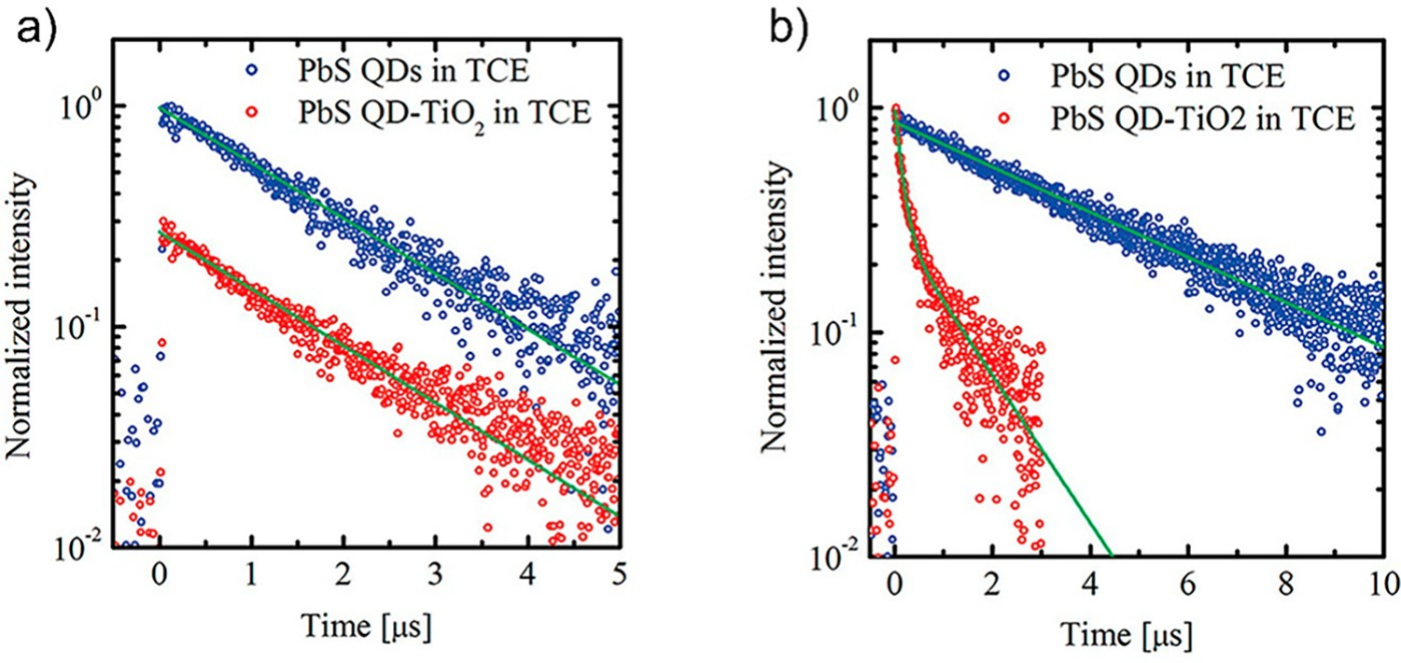
(a) Fluorescence decays of PbS QDs (blue circles) and PbS-TiO_2_ composite (red circles) in tetrachloroethylene (TCE) for PbS QDs with 4.8 nm diameter. For clarity, the fluorescence decay trace of the PbS-TiO_2_ is shifted vertically. (b) Fluorescence decays of PbS QDs (blue circles) and PbS-TiO_2_ composite (red circles) in TCE for PbS QDs with 3.4 nm diameter. Solid green lines: fits using single or double exponential decay functions. Reprinted from ref (153). Copyright 2008 American Chemical Society.

TRPL is a powerful spectroscopic technique that has been employed for resolving electron transfer processes from a QD donor toward an acceptor.^124,132,147−152^ The time resolution of TRPL is limited both by the duration of the laser pulse used for excitation and most crucially by the speed of the employed detector. For relatively slow processes, photomultiplier tubes are used, while streak cameras are preferred for dynamics in the picosecond time scale. Monitoring QD–MO ET by TRPL relies on above-gap excitation of the luminescent QDs and the time-resolved measurement of the PL quenching when the QDs come into contact with the MO, which is expected to act as an electron scavenger. In this respect, the TRPL method employed for monitoring ET at QD–MO interfaces requires obtaining reference decay dynamics of the QD donor species alone. This is typically achieved by monitoring the QD chromophore radiative decay in a diluted solution or cast on an insulating mesoporous MO substrate (e.g., SiO_2_ or ZrO_2_), where interfacial energetics are not suitable for ET (i.e., where the QD-LUMO is energetically placed below the oxide CB). In both approaches, the observed QD reference PL decay rate (*K*_D_) must in principle be equal to the sum of the radiative (*K*_R_) and nonradiative (*K*_NR_) recombination paths within the QDs (*K*_D_ = *K*_R_ + *K*_NR_). Then, a second trace from the QDs sensitizing the MO of interest is recorded. Under the assumption that the sensitization of the MO does not introduce additional recombination pathways competing with ET at the interface or in the QD, the electron transfer kinetic component contributes only with an additional escape route for electrons, with a finite rate *K*_ET_. The measured trace in the QD–MO systems will then provide kinetics defined by *K*_D_* = *K*_R_ + *K*_NR_ + *K*_ET_. It follows naturally that the ET transfer rate from the QD toward the MO acceptor can be inferred as the difference between the two experimental results *K*_ET_ = *K*_D_* – *K*_D_. In many practical cases though, the PL traces after the sensitization cannot usually be modeled by a single exponential function; instead, they require multiple exponential components which are not easy to fully identify and often are assumed to be linked with the chemical heterogeneity and complexity of the surface. An example of the TRPL approach based on measuring QDs in solution and comparing kinetics with those in a QD–MO system is exemplified in Figure 6,^153^ where the work of Hyun et al. is shown for a TRPL experiment to measure electron transfer between PbS QDs (of 4.8 and 3.4 nm diameter) toward TiO_2_ nanoparticles. While larger QDs do not exhibit any change in PL lifetime after sensitization, indicating the absence of ET, for the smaller dots, the authors estimated a fluorescence of the PbS QD decay in tetrachloroethylene (TCE) with a time constant of 4.3 μs, which drops to an average lifetime of 0.7 μs for the PbS-TiO_2_ composite. From these two values, they derived an electron injection time of 0.84 μs for this system.

### 4.2 Transient Absorption Spectroscopy

Likely, TAS has been the most widely used method for characterizing electron dynamics at QD–MO interfaces.^125,126,143,154−157^ After above-HOMO–LUMO QD light excitation with a short femtosecond laser pump pulse, an analogously fast but broadband vis-NIR probe is employed for time-resolving changes in QD absorption as a function of pump–probe delay. The ultrafast, broadband radiation used in TAS is most commonly obtained by supercontinuum generation in a CaF_2_ or sapphire window, which ensures a rather smooth spectrum spanning from the UV to NIR region. The broadband vis-NIR probe spectrum is then directed with reflective optical elements in order to prevent aberrations and is detected with a CCD camera. Negative differential signals in the transient absorption spectra are often associated with charge carrier depopulation events, e.g., of the ground state in favor of the exited states or alternatively originating from stimulated emission. New features that appear instead as positive transients might indicate transitions from the excited levels that are normally not possible when the system is in the ground state.^146^ As such, TAS is capable to selectively monitor the time evolution for the electron population in the QD of each probed state within a broad spectral bandwidth with sub-picosecond resolution.

Like in the case of TRPL described previously, in TAS measurements, the ET process from the QD donor to MO acceptor is often retrieved by subtracting QD related carrier dynamics before and after MO sensitization, or between two QD sensitized MO systems where one of them is made of a wide-gap insulating oxide (typically SiO_2_ or ZrO_2_) with interfacial energetics not allowing ET from donor to acceptor.^126,154,156,158^ In contrast to TRPL, TAS is very powerful owing to the broadband probe employed, that allows one to check not only the ground state bleach but also intraband kinetics within the QD,^93^ an aspect that has been critical for better understanding of QD fundamentals, e.g., the phonon bottleneck effect (as it will be discussed in section 7). Inherent in this approach is the expectation that the dominant electron relaxation pathway after sensitization is indeed the transfer from the QD-LUMO to the conduction band of the MO, which might not be entirely certain. For example, if pump energy excitation does not perfectly match the QD band gap, intrinsic kinetic features as the eventual hot electron transfer from QD high energy states toward the MO-CB or intraband relaxation within the QDs can affect TAS dynamics at early pump–probe delays.

**Figure 7:**
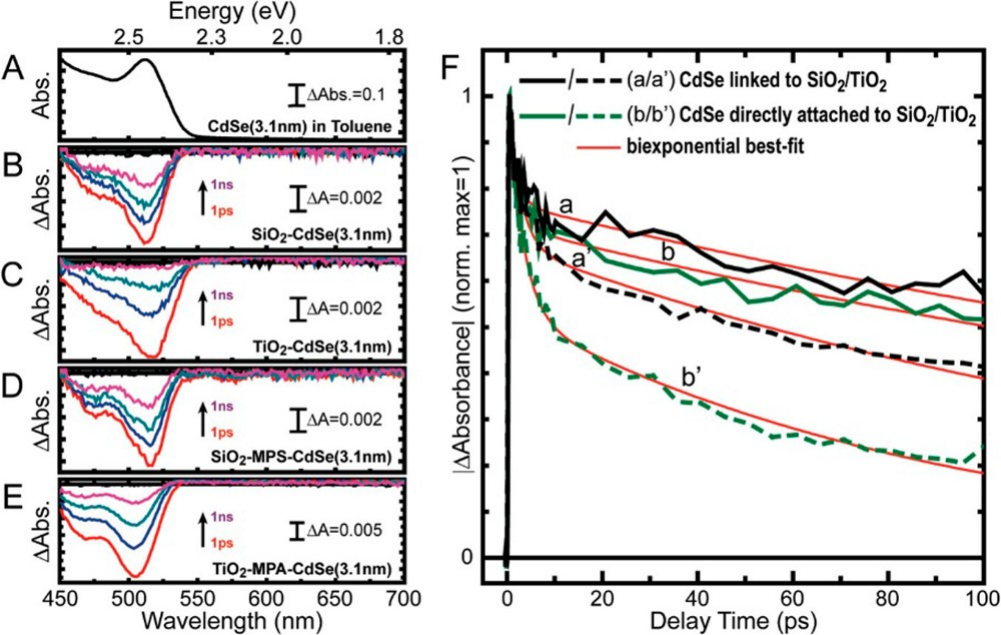
(A–E) Absorbance spectrum of (*d* = 3.1 nm) CdSe quantum dots in toluene solution (A), and transient absorption spectral traces of CdSe QDs attached to SiO_2_/TiO_2_ in a linkerless (B,C) and linked (D,E) fashion. The transient signal decreases with increasing pump–probe delay time: 1 ps (red), 10 ps (blue), 100 ps (teal), and 1000 ps (magenta). Transient absorption kinetic traces of (B)–(E) at the characteristic first excitonic peak of CdSe (F) demonstrate the quenching of the excited state in the presence of the TiO_2_ acceptor. Adapted from ref (159). Copyright 2011 American Chemical Society.

An illustrative example of the TAS methodology made by Pernik et al. is shown in Figure 7.^159^ Following photoexcitation of 3 nm diameter CdSe QDs, the CdSe absorption band associated with the ground state (Figure 7A, in toluene solution) bleaches, which results in the negative transients shown in panels B–E for various sensitizing configurations, i.e., attached to either SiO_2_ or TiO_2_ in a linkerless fashion (B, C) and linked with mercapto-propionic acid MPA (D, E). These transients recover in time as the carriers recombine or are injected to the acceptor. In panel F, the kinetic traces are summarized: because SiO_2_ is electronically insulating and is presumed to not allow electron transfer from CdSe QDs, it is resolved that intrinsic QD relaxation kinetics are measured. By instead attaching the same QDs to TiO_2_, the additional pathway of electron transfer is introduced, which justifies the faster dynamics. With this method, the authors calculate an electron transfer rate constant of *K*_ET_ of 7.2 × 10^9^ s^–1^ in the case of the linkerless adsorption while a slower value of 2.3 × 10^9^ s^–1^ is obtained for the MPA sensitized QDs.

### 4.3 Time-Resolved Terahertz Spectroscopy

**Figure 8:**
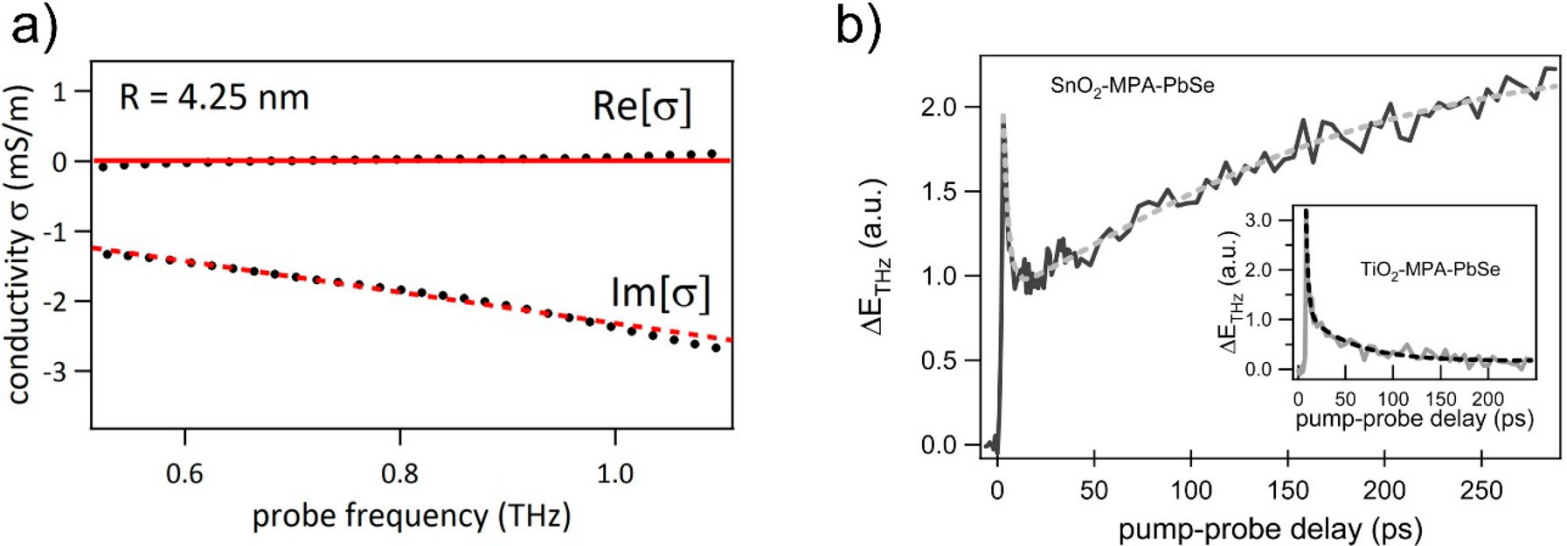
(a) Exemplary THz frequency resolved complex conductivity spectra (black dots) for CdTe nanocrystals in solution showing no real conductivity *Re*[σ] and negative imaginary conductivity *Im*[σ] associated with polarizable excitons in the material. Red solid (real part) and dashed (imaginary part) lines are the results of the best fit to the data by Lorentz model. Reprinted from ref (168). Copyright 2012 American Chemical Society. (b) TRTS signal for the SnO_2_-MPA-PbSe film (4.2 nm PbSe QDs), following photoexcitation with 800 nm pulses. The inset shows a TiO_2_-MPA-PbSe film, characterized by a quasi-instantaneous rise of the signal and a subsequent decay within tens of picoseconds. For the SnO_2_-MPA-PbSe film, there is an additional long-lived ingrowth of the THz signal, which originates from injected electrons in SnO_2_. The dashed lines are fits to the data, yielding a time scale for electron injection of 125 ± 40 ps. Reprinted from ref (30). Copyright 2010 American Chemical Society.

Another powerful method that has been employed to resolve ET at QD–MO interfaces is TRTS.^160^ The pump–probe technique consists of the analysis of pump induced changes of a freely propagating single cycle terahertz (THz) pulse probe transmitted through a particular sample. Typically, few THz bandwidth probes are generated by optical rectification of a femtosecond NIR laser pulse impinging onto a nonlinear crystal such as ZnTe, GaP, or LiNbO_3_.^161,162^ Larger bandwidths reaching few tens of THz can also be targeted with air-plasma sources or spintronic emitters.^163−166^ The THz radiation is often detected in a second nonlinear crystal via electro-optical sampling. Given the low photon energy of conventional THz probes (1–10 THz corresponding to 4–40 meV), THz radiation is unable to trigger band-to-band transitions in most materials; instead, the low photon energy of THz radiation can interact primarily with electrons populating the continuum of states in the conduction band. In this respect, taking into account that the characteristic picosecond time scale of THz oscillations is also comparable to the time scale of charge scattering processes in solids, makes THz radiation a good noncontact optical probe for electric conductivity.^160,167^ Hence, following an optical-pump THz probe (OPTP) scheme, with TRTS, it is possible to selectively excite the QD donor and to probe the finite conductivity of the electrons once they reach the MO acceptor. This is due to the presumed null mobility and hence negligible real conductivity associated with pump-induced neutral excitons populating the QDs (Figure 8a).^168^ On the other hand, the mobility of electrons populating the CB in the MO is finite, which turns measurable pump-induced changes into THz transmission signals. In this respect, TRTS is capable of selectively monitoring electron dynamics in the MO acceptor while dynamics at the QD donor are, in principle, not accessible.^30,126,143,169−174^

As far as we know, the first example of TRTS employed to resolve QD–MO electron transfer was published in 2010 by Pijpers et al.^30^ In Figure 8b, extracted from this work, it is possible to see what can be expected from a TRTS experiment. For an electrode composed of PbSe quantum dots of about 4.2 nm, sensitizing a mesoporous SnO_2_ film by 3-MPA is neatly resolved by an ingrowth of the differential THz signal on time scales of hundreds of picoseconds, which represents the emergence of the real part of electrical conductivity in the samples following above-gap QD photoexcitation. The emergence of the signal unambiguously represents ET taking place from the QD to the oxide matrix. In this case, the common sharp feature appearing at short time delays after photoexcitation in both SnO_2_ and TiO_2_ QD–MO dynamics (inset) was assigned to the formation of QD aggregates in the studied samples, which could also have a nonzero finite electrical conductivity.

The TRTS technique is essentially equivalent to perform TAS with a relatively long IR wavelength as a probe (as was previously employed in dye-MO systems).^24,26^ However, in contrast to both TRPL and TAS, which are techniques based on analyzing changes of photon intensity directly in the frequency domain, in TRTS, it is possible to record pump-induced changes of a freely propagating THz pulse in the time domain.^160^ As such, in TRTS, it is possible to resolve changes not only in the probe amplitude but also in its phase, which in turn gives the advantage of accessing the complex-valued frequency resolved conductivity in a single measurement. Accessing this information enables TRTS to infer independently the mobility of pump induced electrons in the MO by modeling the frequency resolved complex conductivity of a given system.^175−179^

## 5 Experimental Challenges for Disentangling Intrinsic and Extrinsic Kinetic Components

Due to the kinetic complexity taking place at the interface between a QD donor and MO acceptor, it is extremely challenging to univocally quantify electron dynamics at these technologically relevant interfaces. Some technical limitations and several pitfalls associated with them, which can be linked to the misinterpretation of spurious or overlapping signals, together with the fact that QD–MO electron dynamics are extremely sensitive to sample preparation history (e.g., trap population), make direct comparisons between published works at best qualitative, even for very similar systems (e.g., reported ET rates spanning 5 orders of magnitude for, apparently, “the same” lead salt-TiO_2_ QD–MO systems).^30,153,180−182^ These issues make it rather difficult to perform differential analysis on these systems, i.e., explore the interfacial dynamics as a function of only one variable. This is illustrated in much detail in section 6, where we present a summary of fundamental studies analyzing ET as a function of key parameters as the coupling strength or the excess energy between donor and acceptor states (Δ*G*). In the following, we will comment on some of the most common experimental challenges that one can encounter when measuring ET on QD–MO systems within the framework of the three main techniques discussed in the previous section; these issues can be grouped as those linked to method and methodology and those linked to sample history and photostability.

### 5.1 Method and Methodology

Almost all techniques considered above rely on the subtraction/comparison of experimental data sheets for obtaining ET rates at the QD–MO interface. Data sheets are taken under often very dissimilar conditions; in this respect, one major hurdle is to ensure in any report that the subtraction/comparison of data sheets toward resolving the ET component of interest is feasible.

Let us start by mentioning that both TRPL and TAS are primarily sensitive to the dynamics from the point of view of the QD donor (pump the QD and probe the QD). As such, dynamics linked with charge carriers in the MO acceptor are, in principle, not accessible. On the other hand, TRTS (or TAS with a far-IR probe) is primarily sensitive to the dynamics from the point of view of the MO acceptor (pump the QD and probe the MO acceptor). In this case, dynamics in the QD are not directly accessible. The presumed pump–probe selectivity in the experiments can be very misleading when trying to interpret the data if is not taken with enough care. For example, often TRTS reports on ET studies do not mention a differential approach (as TAS and TRPL do) consisting of the subtraction of kinetics for, e.g., QDs in solution and QD–MO samples. The reason is that authors assume a priori that excitons populating the QD in the QD–MO system are not delivering any signal in a vis-pump THz-probe spectrum. This seems very reasonable taking into account that a TRTS measurement should resolve only free carriers in the QD–MO system, which can in principle only populate the oxide CB, and not the QD, where only neutral excitons should be present (Figure 8).^30,160,168^ However, this is not entirely true in all cases, as colloidal QD suspensions can produce a finite conductivity in some occasions. For instance, reports indicate a loosening of quantum confinement depending on the QD size,^168^ that can eventually produce a finite conductivity within the ∼2 THz probe window generally employed in experiments. Furthermore, the THz probe could be sensitive to direct intraband transitions within the less sparse QD hole states.^97^ In this sense, it is always good practice to measure the QD THz response in solution prior to analyzing the QD–MO system. Nevertheless, even by taking into consideration these measures, one can still face challenge; i.e., even having a null signal from the reference measurement of QD in solution, one might wonder whether the same response is expected for a QD in contact with the MO. For example, the QDs can aggregate in clusters with a finite conductivity, which can be promoted by bottlenecks in the pores of electrode (as we discuss in section 5.2). Alternatively, upon ET toward the MO conduction band, the neutral exciton populating QDs in solution is dissociated at the interface and now the remaining hole in the QD could eventually undergo THz intraband transitions within the QD hole states, giving rise to a finite TRTS response.

Most of the comments made above about eventual pitfalls assumed by probe selectivity linked to TRTS data interpretation are readily applicable to TAS and TRPL methods. These methods always employ a methodology based on differential analysis, i.e., by the subtraction or comparison between kinetic traces taken in QDs in solution (or QD-SiO_2_/ZrO_2_) vs the QD–MO of interest. The selectivity in the probe in TRPL is often valid, as after QD excitation one can resolve a relatively narrow spectral region where radiative decay of the QD takes place. This emission is easily identifiable on the spectrum, and one should not expect radiative emission signals to overlap this one from any other spurious source at the QD–MO interface. On the other hand, the decay mechanism in TRPL analysis can be very complex, involving many different deactivation pathways that can easily complicate the true identification of the ET component within the data simply by comparing it to a reference sample. Typical sources of additional complexity to the decay in TRPL can be traps in the QDs, traps in the MO, the presence of QD aggregates, etc., which could in principle change the nature of radiative relaxation in the absorber before and after functionalization of the MO by the QDs.

The emergence of spurious signals in ET dynamics at QD–MO interfaces can also affect the TAS method. One main advantage of TAS is the possibility to probe a broad UV–vis spectrum at once. On the other hand, this broad spectrum can be composed of many kinetic and interrelated components acting at the same time on the probed spectral window. The nature of this problem is obviously largely dependent on the nature of the QD under study.^157^ For example, ET from CdX (X = S, Se, Te) QDs to TiO_2_ can be easily discriminated from TAS data due to the fact that the signal is dominated by the state filling of the 1S electron level.^52,183−186^ However, in PbS QDs, both 1S electron and hole states contribute to the overlap of transient absorption features (1S exciton bleach and induced absorption) at the same time in the same spectral region. The direct consequence is that the contribution to the overall signal cannot be easily assigned to either species.^30,187^ Judiciously selective probing by TAS of different spectral regions can be done for disentangling these kinetic components.^157^ This overlap of signals and the linked lack of probe selectivity is especially relevant at early pump–probe delays and when using a pump energy in high excess from the HOMO–LUMO gap (i.e., under nonequilibrium conditions, rather than quasi-steady-state). Finally, analogously to the cases described for TRPL and TRTS, typical sources adding complexity to the decay in TAS spectra can be linked to the presence of traps and aggregates providing spurious kinetic fingerprints that could be identical in line shape to the one expected for ET from the QD toward the MO.

The presumed selectivity in the probe for all of the different methods discussed above should also be accompanied by, ideally, a selectivity in the pump. In order to monitor unambiguously ET from the LUMO to the oxide CB, an ideal situation is to perform experiments with a pump energy precisely matching that of the HOMO–LUMO QD gap. In fact, by doing so, one will prevent hot carrier effects taking place at the QD–MO interface, i.e., hot carrier effects that can mask the signal of interest. Effects related to hot carriers, e.g., thermal relaxation or multiexciton generation and associated Auger recombination, typically take place within few picoseconds to hundreds of picoseconds, respectively, after the QD light excitation.^93,188^ As such, they will primarily affect early pump–probe delay dynamics in QD–MO systems. Regarding pump selectivity, note that a typical 400 nm excitation pump has enough energy to produce band-to-band transitions in TiO_2_ and ZnO electrodes.^14,15^ Furthermore, if the oxide presents midgap donor states of any kind,^2^ these can be eventually pumped by below-gap pump photon. These signals will produce spurious kinetic components that can misleadingly be assigned to an ultrafast ET process from the QD to the oxide. Therefore, a separate analysis of the response of the oxide alone should be part of every experimental routine.

Apart from the excess energy selectivity discussed above, one should attempt measuring charge carrier dynamics in the linear excitation regime, i.e., under excitation conditions validating single exciton dynamics per QD. Only if data is collected for reference QD and QD–MO systems under linear conditions, one can guarantee a fair subtraction or comparison. Even taking into account these considerations, photocharging effects can affect the dynamics of QDs in solutions.^45,189^ To overcome these issues, one can stir the samples during measurement. However, the same procedure cannot be done in a QD sensitized mesoporous film, adding complexity to the problem.^190^ Most of these effects can be discriminated by performing photon fluence dependence analysis and, as stated above, validating that charge carrier dynamics are invariant as a function of the number of incident photons.

### 5.2 Sample History and Photostability

In addition to the methodology aspects discussed above, QD–MO interfacial dynamics could be extremely sensitive to sample preparation history. For example, a different amount or nature of traps in the QDs will substantially affect the pump induced dynamics monitored in one system. However, the relative impact of trapping on determining ET rates can be method-dependent: while trapping in the QD might not be detected by TRTS (a trapped electron does not provide a finite conductivity), it would critically affect TAS and TRPL line traces, where the kinetic fingerprint of trapping (an exponential component) will be the same as the one associated with ET. The comparison of the kinetics of QDs in solution (or, e.g., QD-SiO_2_) and QD–MO should in principle remove this component differentially, under the assumption that sensitization of the MO by the QDs does not produce new or more traps competing with ET.

As stated previously, another source that might affect the monitored interfacial kinetics obtained by TRPL, TAS, and TRTS methods is the potential presence of QD aggregates within the mesoporous oxide film. Several groups reported the effect of aggregates on TRTS dynamics. They showed that the aggregation of QDs in QD–MO samples enables the delocalization of electrons within QD aggregates.^169,170,172,191^ These aggregated phases can result in TRTS dynamics as short-lived kinetic components, as it happens in QD superlattices that present a finite electron conductivity.^180^ In this line, Wang et al. correlated kinetics and high resolution TEM analysis as a function of QD loading^169^ and showed that a lack of aggregates produced TRTS ET dynamics that were perfectly defined by a single exponential function for excitation near the band gap.^170,172,173^

We need to consider another additional issue, linked to the eventual presence of MO surface states that may act as electron scavengers after sensitization. Indeed, such loss channels and the associated breakdown of correlation between optical signatures and ET have been identified, e.g., for sensitized ZrO_2_ electrodes,^30,123,124^ a system where ET from the QD-LUMO to MO-CB is energetically prohibited (i.e., the MO-CB lies energetically above the QD-LUMO state). These pathways could also be present in state-of-the-art electrodes (TiO_2_, ZnO, and SnO_2_), as already suggested by some authors.^125−127^ However, given that the spectroscopic signature of ET to the MO-CB and those associated with recombination and trapping processes at the interface are in both cases exponential functions, it is quite challenging to differentiate between them by measuring depopulation kinetics by TAS or TRPL from the QDs. Hence, observing the disappearance of charge carriers from the QD after sensitization alone may not unambiguously determine whether the carriers are actually injected into the oxide conduction band or vanished along another path induced by the MO sensitization. On the other hand, the specific defects present in a given MO will also differ depending on the way the oxide has been produced and handled.^13,17,18^ The size of the MO particles, exposed crystalline facets, and specific chemistry will determine critical aspects, such as the relative position of the Fermi energy in the MO relative to its CB. This alone will largely determine QD–MO interfacial energetics (see section 6.1) and hence the monitored ET dynamics at that interface.

**Figure 9:**
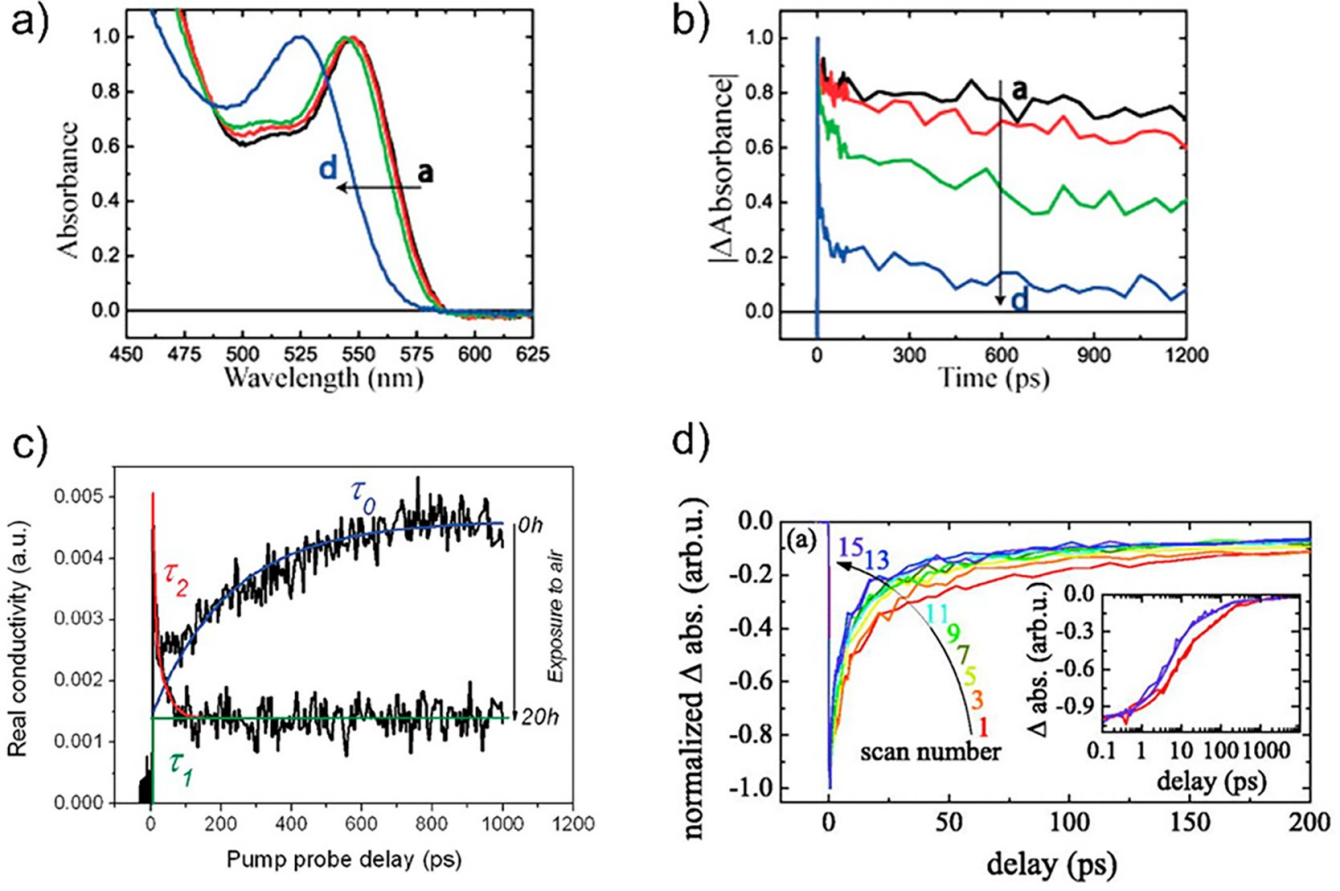
(a) Normalized absorption spectra and (b) bleaching recovery kinetics measured via femtosecond transient absorption spectroscopy at the ground-state bleach maximum (548–523 nm) of a CdSe QD suspension in toluene following UV irradiation and air exposure: (a) 0 h, (b) 1 h, (c) 3 h, and (d) 20 h. Adapted from ref (117). Copyright 2012 American Chemical Society. (c) Characteristic carrier dynamics monitored by THz-TDS on PbSe QDs sensitizing SnO_2_ film before (0 h) and after (20 h) exposing the sample to air. The highlighted time constants (τ_0_, τ_1_, and τ_2_) are correlated with three different mechanisms. Reprinted from ref (172). Copyright 2011 American Chemical Society. (d) Normalized TAS kinetics under femtosecond laser irradiation in low O_2_ atmosphere measured in sequence. Inset: first (red line) and last (violet line) scan of TAS decay (thin lines) fitted by a three-exponential decay (thick lines). Reprinted from ref (193). Copyright 2012 with the permission of AIP Publishing.

While surface defects in QDs are likely ubiquitous in any experiment, a known way to identify their kinetic fingerprint and eventually correlate it with the nature of the promoted defects has been to analyze charge carrier dynamics under controlled photo-oxidation for colloidal suspensions.^117−120^ In simple terms, photo-oxidation of colloidal dots promotes ultrafast trapping evidenced as a clear quench in TRPL and TAS associated dynamics (see Figure 9a,b). The trapping is then linked with the generation of oxide species at the QD surface. Additionally, in the long term, a shrink in the effective QD size can be experimentally resolved as a band gap widening.^117,119^

Photostability issues in the QD–MO samples during measurement can be prevented by the encapsulation of the samples under inert environments, typically under dry N_2_ atmosphere or vacuum. However, some authors have taken advantage of a controlled photo-oxidation of the samples to distinguish between ET and other spurious signals contributing to interfacial dynamics.^122,170,172,192,193^ For example, by purposely exposing a PbSe QD sensitizing SnO_2_ (Figure 9c) sample to air during pump–probe data collection, Cánovas et al. revealed by TRTS that two out of three observed kinetic components contributing to the data persisted following full photo-oxidation of the samples.^172^ The kinetic fingerprint of ET purely vanished (as expected from favoring the competition between QD surface trapping and ET toward the oxide), while the other two spurious components remained invariant upon sample degradation. Another example with TAS was studied by Žídek et al.,^193^ where controlled photo-oxidation revealed that all the observed components of TA kinetics, which reflect electron dynamics in CdSe, are affected by photodegradation, leading to a faster TA decay (Figure 9d). In addition, they observed a prominent superlinear dependence of the TA photodegradation rate on femtosecond-laser irradiation intensity. They used this information to estimate the back-recombination time of electrons injected to ZnO.

Finally, most of the studies that have been taken into consideration in the literature (and in this Review) measure ET in systems that consist only of the QD-donor/MO-acceptor systems. Several authors gave evidence of the importance of determining kinetics in an environment as close as possible to the one seen in a device of interest. In a complete working device, either a photovoltaic or a photocatalytic cell,^138,139^ there is the necessity of a hole selective contact that harvests the positive charges that otherwise remain localized in the QD after the ET event toward the MO. Makarov et al.,^190^ for example, identify from TRPL measurements how the lack of a hole scavenger in the sample, required in a working solar cell, leads to the presence of long-lived photoexcited holes in the QDs. The permanence of charged species indeed introduces artifacts, which they attribute to the formation of fast positive trion Auger decay. According to the view of the authors, this effect can dominate electron dynamics and mask true ET as seen by TRPL.

## 6 Fundamental Studies of Electron Transfer at QD–MO Interfaces

**Figure 10:**
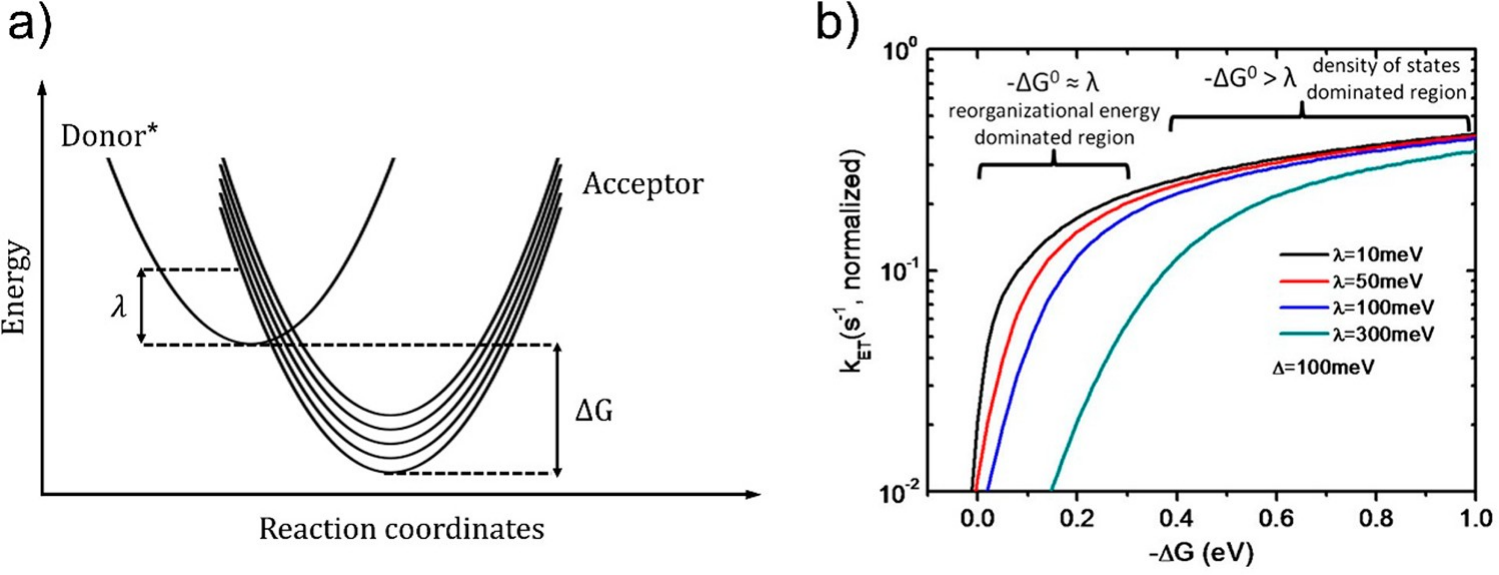
(a) Schematic of the energetics of a general ET process. λ denotes the configurational energy needed to overcome the barrier between the donor in the excited state (Donor*) and a manifold of accepting states (Acceptor). Δ*G* is the free energy difference between the ground levels of donor and acceptor. (b) Dependence of *K*_ET_ vs Δ*G* for various reorganizational energies λ in a metal oxide nanocrystal with Gaussian-shaped band edge defects of width Δ = 100 meV. Reprinted with permission from ref (154). Copyright 2011 National Academy of Science.

Generally speaking, the model describing the rate of electron transfer between a donor and acceptor that is commonly accepted is the one initially introduced by Marcus and further developed, among others, by Gerischner.^194−196^ In the nonadiabatic limit, the thermally induced reorganization of the involved species and their surroundings is what creates a favorable arrangement for the ET process to occur. In the past, electron transfer at dye sensitized MOs has been interpreted within the nonadiabatic Marcus theory.^26^ For the case of QDs sensitizing a MO surface, the same theoretical background has often been assumed, and indeed proposed, to be governing interfacial dynamics.^154^ In this case, electron transfer from a single QD state takes place toward the CB continuum of accepting states that characterizes the sensitized MO surface. Under these conditions, the so-called many-states Marcus formalism takes the form:
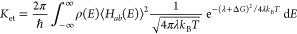
1Here, Δ*G* refers to the Gibbs free energy variation, λ is the reorganizational energy, *T* is the temperature, and ρ(*E*)⟨*H*_*ab*_(*E*)⟩ represents the density of accepting states in the MO multiplied by the coupling strength between initial and final states (which in eq 1 is integrated over all the potentially available accepting states). In simple terms, the free energy change is the energy difference between the donating state and the bottom of the acceptor conduction band. The reorganizational energy (both inner and outer sphere components) includes all structural changes in the reactants and the environment during charge transfer. Generally, a plot of K_*et*_ vs Δ*G* will show a steep rise at energies Δ*G* ∼ λ and a gradual increase at energies Δ*G* > λ, the regions where transfer dynamics are dominated by the reorganizational energy and the density of electron accepting states, respectively (see Figure 10).^26,154^

Although monitoring tunneling via molecular conductance between two metal contacts and electron transfer in D–bridge–A systems might seem rather different, Nitzan has proposed that both mechanisms are directly proportional when the bridge operates as a simple resistor to current flow (obeying Ohm’s law).^50,86,197,198^ The ET rate is expected to have an implicit dependence on the distance between the D–A pair that depends essentially on the nature and the magnitude of the electronic coupling term.^86^ When the coupling β is strong, it is expected that ET is governed by a coherent tunneling process with a typical exponential dependence on the donor–acceptor distance (*d*):
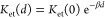
2A transition from tunneling to a hopping mechanism will happen as the donor-to-acceptor distance is increased. The two regimes of tunneling and hopping can be discriminated in principle via temperature-dependent analysis, as a coherent tunneling process does not depend on temperature while hopping, on the other hand, requires an activation energy usually provided by the thermal bath.^198,199^ Hopping requires available electron sites populating the barrier; in this case, the electron transferred from the donor to the acceptor may actually reside on the barrier for a certain amount of time and may hop between localized sites on the bridge itself. In this limiting scenario, ET will be barely affected by the distance between the donor and acceptor, as ET will be defined by the last hop event from the barrier to the acceptor state. In general, both mechanisms are operative at the same time, and a temperature analysis can show which of them is dominant.

In the following, we will attempt to critically present some of the relevant literature aiming at addressing the fundamentals of ET at QD–MO interfaces. Specifically, we will introduce results that analyze the dependence of ET rates on the key parameters contained in eq 1.

### 6.1 Effect of Δ*G* on ET

**Figure 11:**
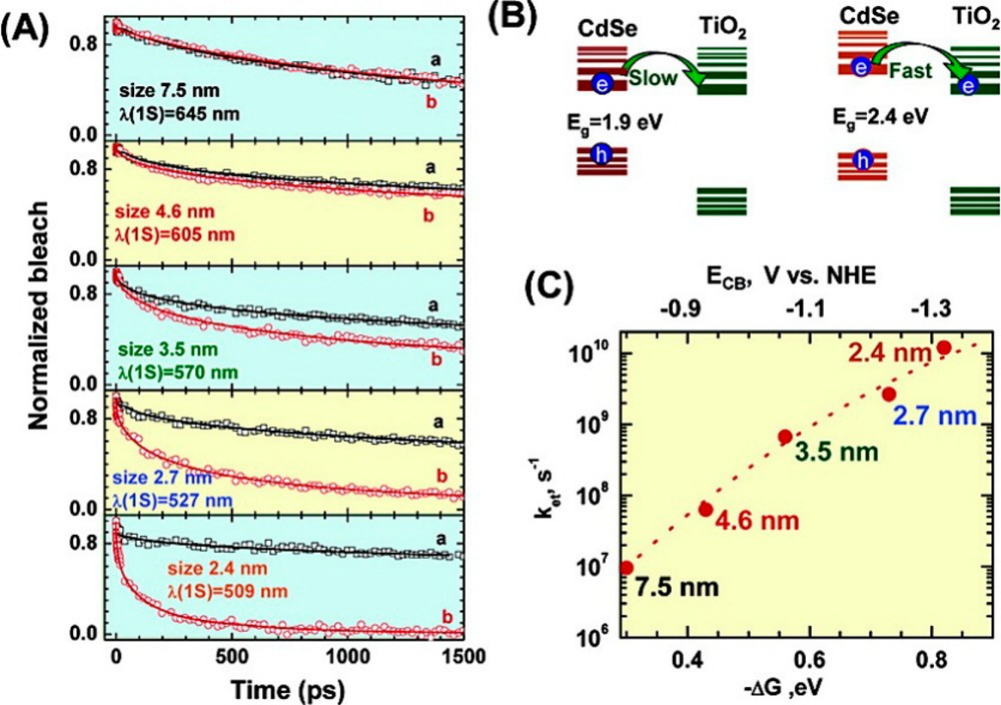
(A) Transient recovery recorded by TAS at the bleaching maximum following 387 nm laser pulse excitation of CdSe quantum dots in 1:1 ethanol/THF containing mercaptopropionic (MPA) acid (a) without and (b) with linked TiO_2_ particles. (B) Scheme illustrating the principle of ET from quantized CdSe into TiO_2_ and (C) the dependence of ET rate constant on the energy difference between the QD-LUMO and the bottom of the oxide CB. Top axis represents assumed CdSe conduction band energy positions vs NHE. Reprinted from ref (156). Copyright 2007 American Chemical Society.

The tunability of the QD band gap via nanocrystal size has been employed by several groups to analyze the dependence of ET rates on D–A excess energy (Δ*G*) at QD–MO interfaces.^38,126,131,154,156,172,200,201^ To our knowledge, the first report that specifically aimed to analyze ET vs Δ*G* was made by Robel et al. by TAS, who studied CdSe quantum dots of various sizes, ranging from 7.5 to 2.4 nm, sensitizing relatively large (40–50 nm) TiO_2_ nanoparticles in suspension (Figure 11).^156^ In this work, the authors compared dynamics of QD in solution with QD chemically attached by MPA to TiO_2_ nanoparticles, and demonstrated an almost exponential dependence for ET rates with the energy difference between the LUMO of the donor and the CB of the TiO_2_ acceptor. This result is qualitatively in line with the prediction of Marcus theory (given in eq 1), however the limited range of energies that can be analyzed by modifying QD band gap (i.e., by quantum confinement) undermines the possibility to explore ET rates vs Δ*G* over a wider range of energies, a requirement for performing a reliable fit to the Marcus model.^202^

**Figure 12:**
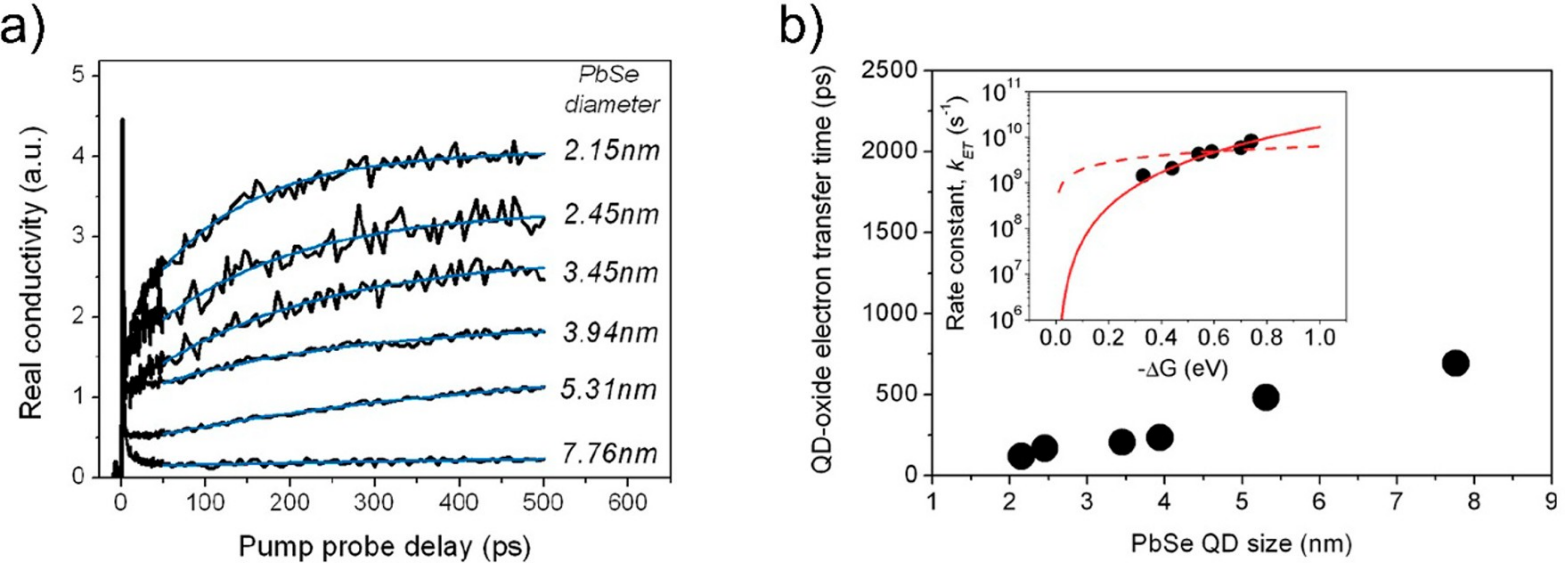
(a) QD size-dependent electron transfer monitored by TRTS on PbSe QD sensitizing SnO_2_ films. Blue lines show a single exponential increase in the conductivity. (b) QD size-dependent electron transfer lifetime for PbSe dots anchored to SnO_2_ by MPA. The inset depicts the corresponding rate constants as a function of the relative band alignment (Δ*G*) between donor and acceptor species; the solid (and dashed) red line is the best fit following many-states Marcus theory when considering a quadratic (or constant) dependence on energy for the wave function overlap between the QD and the oxide. Reprinted from ref (172). Copyright 2011 American Chemical Society.

Cánovas et al. revealed a similar dependence on PbSe QD sensitizing mesoporous SnO_2_ with MPA (Figure 12).^172^ By employing TRTS, they directly showed a signal increase after pump arrival, ascribable to QD–MO ET, which was modulated by QD size with a larger rate constant for smaller QD. The authors assigned the modulation of ET as a function of size to the variation of Δ*G*, defined as the donor–acceptor energy difference estimated form the relative energy level position versus the vacuum level of isolated constituents. Their results were also explained within the many-states Marcus theory (eq 1). However, these findings again are limited to a small range of Δ*G* values which does not allow one to explore accurately the predicted trend for ET as a function of Δ*G* as shown in the inset of Figure 10b.

Žídek et al. reported an analogous result on colloidal CdSe QD sensitizing ZnO nanowires via the bifunctional molecule 2-mercaptoproprionic acid.^126^ A merit of this work lies in the combination of TAS and TRTS to resolve neatly the ET process of interest. With the combination of the two techniques and by employing CdSe QDs with sizes between 2.5 and 3.1 nm, the authors conclude rate trends with Δ*G* that once more, in this case for ZnO, seem to follow qualitatively the predictions of the Marcus model. In any case, the fit to the data is again very limited in the Δ*G* axis to unambiguously conclude whether the theory fully applies.

In order to address the fundamentals and to bypass the limitations in accessible Δ*G* given for a QD–MO system as a function of QD size, Tvrdy et al. reported TAS ET rates ranging from 10^10^ to 10^12^ s^–1^ for CdSe QD sizes sensitizing TiO_2_, SnO_2_, and ZnO MO electrodes.^154^ By employing different oxides with distinct work functions, they attempted to widen the range of energies interrogated. Their results were globally fitted to the many-states Marcus model while assuming the same QD–MO coupling term valid for every employed MO. This approach is arguable as each metal oxide will provide a distinct and unique coupling term, and then one could expect that a single global fit to the whole data is not feasible.

In all of the works discussed above, an exponential dependence between the ET transfer rate and the donor–acceptor excess energy Δ*G* is found for the most scrutinized MOs, i.e., TiO_2_, SnO_2_, and ZnO, even when employing different methodologies.^126,156,172^ However, as previously discussed, given the lack of dispersion in the provided data, one could only conclude in all cases that a qualitative agreement with Marcus theory exists. To complicate things a bit further, all of the works discussed above provide Δ*G* estimates from the difference in work functions determined from isolated donor and acceptor entities, i.e., following the assumption that isolated work functions are preserved for the QD–MO assemble (i.e., assuming weak coupling at the interface).^126,154,156,172^ However, many studies with QD–MO and QD–bridge–MO systems have proven that Δ*G* estimates obtained in this way are likely not reliable. In fact, when the donor–acceptor coupling is strong, wave function mixing and pinning effects can produce very small (even negligible) modulations of Δ*G* vs QD size.^203,204^

**Figure 13:**
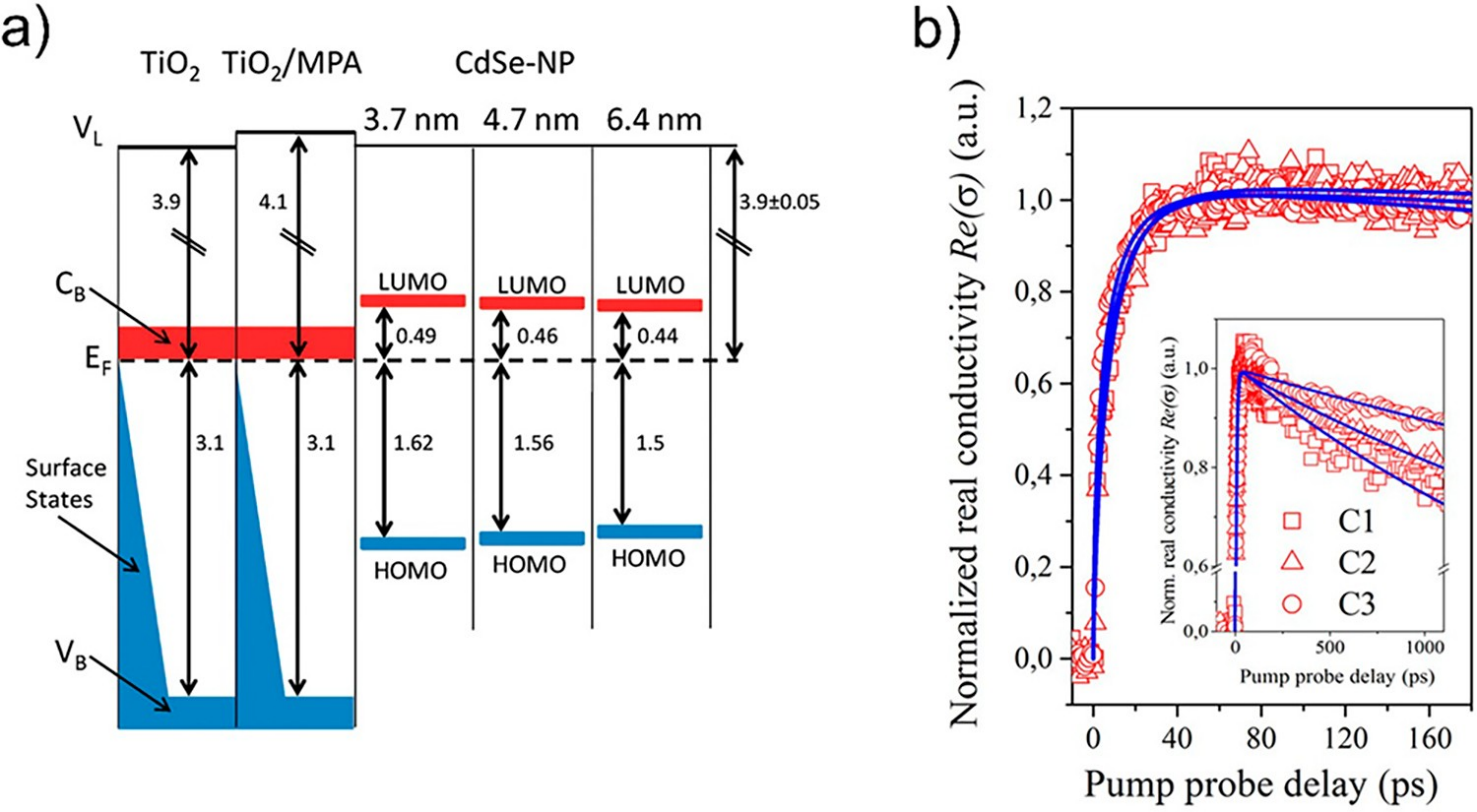
a) Energy diagram for ITO/TiO_2_ and ITO/TiO_2_/MPA and when CdSe QDs are adsorbed ITO/TiO_2_/MPA/CdSe-QD, for three different QD sizes. Both the occupied electronic states (blue) and the unoccupied electronic states (red) are shown. As the size of the QDs increases, the HOMO–LUMO energy gap decreases and the shift of the HOMO is ∼2.4 times larger than that observed for the LUMO. Reprinted from ref (203). Copyright 2011 American Chemical Society. (b) Normalized OPTP response for PbS QDs sensitizing SnO_2_ as a function of the number of SILAR cycles (C_*n*_, with *n* = 1–3); in the inset are shown the dynamics up to 1.1 ns, illustrating the back electron transfer that occurs at longer time scales; blue lines are best fits to the data. Adapted from ref (169). Copyright 2014 American Chemical Society.

Some examples in which the interfacial energetics of the D–A system show near-pinning or pinning conditions are shown in Figure 13.^169,203,204^ Markus et al. employed optical spectroscopy, low-energy photoelectron spectroscopy, and two-photon photoemission to measure the relative band alignment of CdSe QDs linked by MPA to TiO_2_,^203^ revealing a rather small Δ*G* modulation vs QD size. Note that for the range of QD sizes analyzed one can infer a variation in Δ*G* of above 200 meV when considering the work function of isolated systems; in the real system, however, the variation is instead about 50 meV, four times smaller. In another work analyzing ET rates from QDs grown by SILAR onto SnO_2_, Wang et al. showed that the injection rate can be even invariant with the size of QDs, while QD size indeed drastically affects the back electron transfer component (BET, see Figure 13).^169^ This observation was rationalized by pinning at the interface. Here, electron injection from the QD-LUMO to the oxide CB is size-independent because *E*_LUMO_ – *E*_F_ is nearly identical in every sample. BET, on the other hand, increases in smaller dots because the energy difference between oxide CB bottom and QD-HOMO increases as QDs are reduced in size. The pinning at this specific interface was verified in a follow up work from the same group via ultraviolet photoelectron spectroscopy studies (UPS).^204^ It is therefore of pivotal importance to determine experimentally the energetics at the interface for a better estimate of the Δ*G* for any donor–acceptor system.^205^

In order to tune the energetics over a wider range of values without the need to change the MO nature or the QD size, one can attempt to modulate the QD work function by employing organic molecules with variable dipole moments to decorate the surface of the QDs.^204^ Several works with QD solids have indeed demonstrated that a “QD capping” corona of molecular dipoles was very effective for tuning the work function of QDs (up to 21.7 meV/Debye).^72,73^ On the other hand, several reports made on QDSSC architectures, where a similar dipolar capping of the QDs was employed, revealed a null effect on modulating *V*_oc_.^206,207^ Based on the combined analysis of interfacial dynamics at QD–MO interfaces by TRTS and interfacial energetics by UPS, Wang et al. explained this apparent contradiction by revealing a lack of work function modulation induced by “QD dipolar capping” due to Fermi level pinning at the strongly coupled QD–MO interface. This conclusion does not mean that the idea of employing dipolar capping to control the Fermi level and the relative band alignment is unfeasible at QD–MO interfaces, but it would require the prevention of the Fermi level pinning at the interface. To achieve this condition, a possible strategy is to reduce the QD–MO coupling strength by the insertion of an additional decoupling layer (e.g., an insulating metal oxide) between the donor and acceptor as demonstrated by Bloom et al.^208^

An additional example of Δ*G* modification without using QDs of different size was employed in the work of Chakrapani et al.^155^ In this work, the authors observed a modulation of ET in a colloidal CdSe-TiO_2_ system as a function of the pH of the solution in which the system was immersed. The transfer rate constant was shown again to depend exponentially on the Δ*G* between the donor and acceptor. According to the authors, this modulation was linked to a pH-induced protonation of TiO_2_ surface groups, which is capable to shift the band edge of the MO.^209^ On the other hand, CdSe passivated with hydrophobic functional groups such as the employed trioctyl phosphine oxide (TOPO) was useful as it renders the QD semiconductor surface insensitive to pH. The results showed once more a trend in qualitative agreement with Marcus theory.

### 6.2 Effect of Coupling Strength on ET

The impact of QD–MO coupling strength on electron transfer has been studied experimentally by rationally engineering the interface between the donor and acceptor, e.g., by modifying the nature of the bridge, its length, or the chemistry of its head groups.^28,158,170,182,210−215^ When a molecule is employed as a bridge for promoting the anchoring of QDs to the MO surface, the system is typically referred as a donor–bridge–acceptor (D–B–A) system. The molecular bridge serves as a docking site for the functionalization of the MO surface; this is achieved by employing linkers with bifunctional head groups. Typically, one end of the molecule possesses a carboxylate (−COOH) that links preferentially to the MO surface, and the other end of the molecule has, e.g., a thiol group (−SH), which possesses a strong chemical specificity to the metal atoms in the QDs (e.g., lead and cadmium chalcogenide QDs, PbX, and CdX, respectively, with X = Se,S,Te). The bridge, apart from promoting QD docking, plays an important role electronically, as it introduces an insulating barrier between the donor and acceptor. This aspect is clear from most of the works that have reported changes in ET rates from the QD to MO by changing the interfacial chemistry between the donor and acceptor.^28,158,170,182,210−215^ However, in order to better understand the electronic-electric role of the molecular bridge and its impact on ET rates, it is of primary importance to design experiments where only one key parameter in the imposed insulating barrier is modified carefully at the time, e.g., the D–A distance is changed while keeping the energetic height of the insulating barrier unperturbed. As far as we know, Dibbell and Watson^210^ were the first to approach critically the question on how ET rates are affected by the QD–MO distance. They studied CdS-TiO_2_ linked by bifunctional mercaptoalkanoic acids of various chain lengths (by extending the length of the molecular bridge barrier by adding CH_2_ groups). They employed a combination of TRPL and TAS and unambiguously revealed a neat impact on ET rates induced by changes in the bridge nature. A similar attempt was made by Hyun et al. with TRPL on PbS–-bridge–TiO_2_ systems, also showing a neat modulation of ET rates as a function of the length of the bridge settled by the number of CH_2_ groups.^182^ However, in both works, the expectation of resolving an exponential decrease in rate constants vs bridge length (see eq 2 herein) was not achieved experimentally.

**Figure 14:**
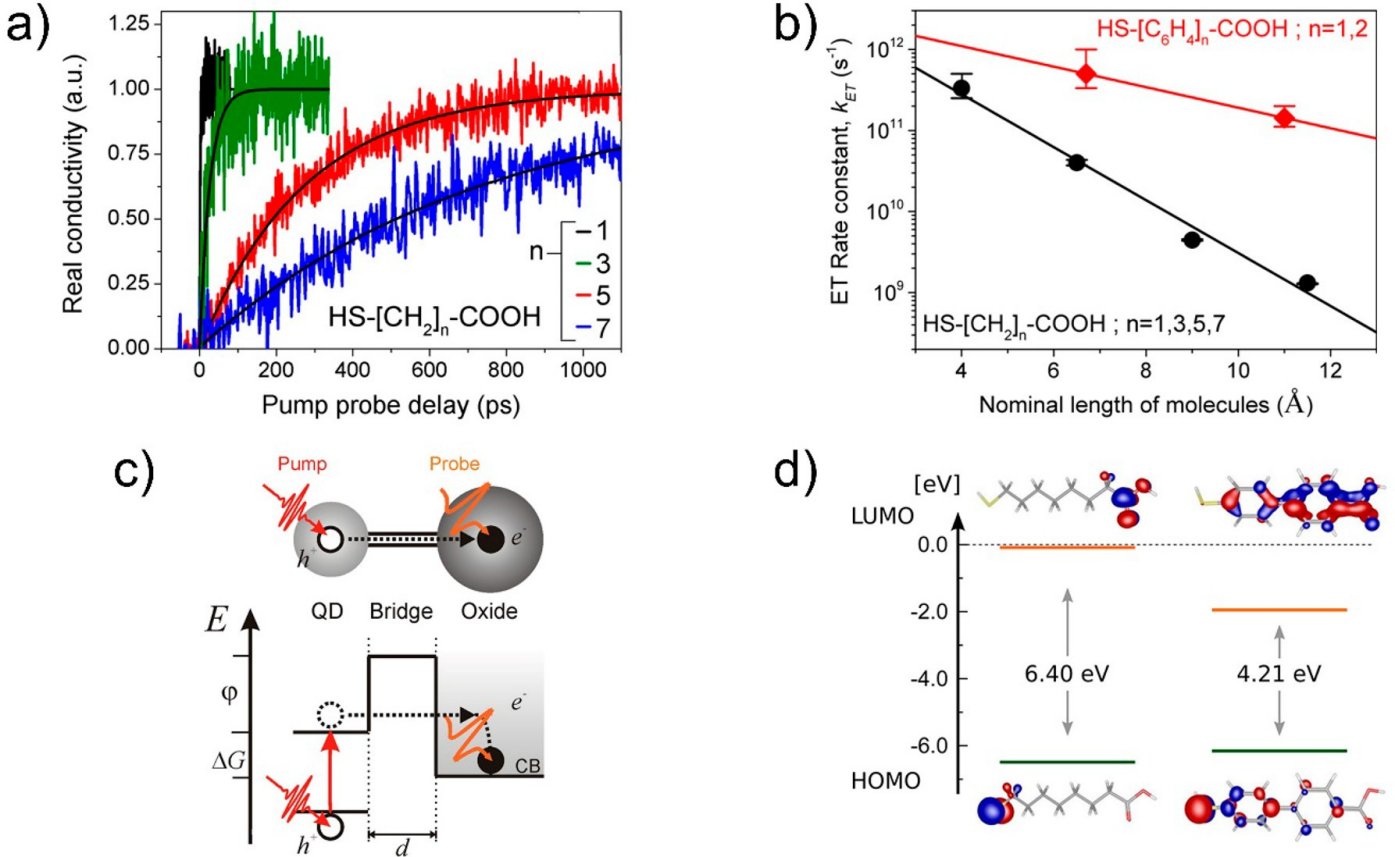
(a) ET kinetics between ∼3 nm CdSe QDs and SnO_2_ through *n*-methylene based bridges (HS–[CH2]_*n*_–COOH, with *n* = 1, 3, 5, 7. Black lines depict single exponential fits. (b) Estimated ET rate constants vs molecular bridge length for *n*-methylene and *n*-phenylene based bridges. Solid lines are best fits to an exponential dependence with the distance. (c) Principle of operation for time-resolved THz photoconductivity measurements and sketch of the energetics of the QD–bridge–oxide system. The bridge acts as a tunneling barrier between the QD and oxide. (d) Calculated energetic and spatial distribution of the frontier orbitals of HS–[CH_2_]_7_–COOH (left) and HS–[C_6_H_4_]_2_–COOH (right) molecules. The aromatic bridges show a reduced barrier height. Adapted from ref (170). Copyright 2013 American Chemical Society.

Following a similar approach, Wang et al. used TRTS to study the effect of bifunctional *n*-methylene (SH–[CH_2_]_*n*_–COOH) based molecules on ET rates when used as molecular bridges between CdSe QDs sensitizing SnO_2_ (Figure 14).^170^ In this case, the article shows neatly how the ET rate decays exponentially with bridge length for the case of *n*-methylene based molecules (Figure 14a,b); the data can be modeled by eq 2, where the term β contains information about the “shape” of the tunneling barrier β = −(2/*a*) ln(*H*_bb_/Δ*E*_db_), where *H*_bb_ is the internal coupling energy between bridge units, *a* is the bridge unit length, and Δ*E*_db_ is the energy of the mediating tunneling state above the donor ground state. This work demonstrates that the bridge acts as an insulating barrier toward current flow and more importantly reveals that coherent tunneling is the dominant mechanism determining the ET rate between the donor and acceptor (Figure 14c). The authors also analyzed the impact of having a reduced barrier height between the donor and acceptor by using *n*-phenylene (SH–[C_6_H_4_]_*n*_–COOH) molecules, i.e., keeping the same head groups but changing the nature of the backbone. They demonstrate that, for a given QD donor-oxide acceptor separation distance, the aromatic *n*-phenylene based bridges allow faster electron transfer processes when compared with *n*-methylene based ones, in line with a reduction of tunneling barrier height for aromatic rings compared to aliphatic chains (Figure 14d). It is worth noting here that the observation of an exponential decay on ET rates vs molecular backbone length is only achievable if an extra molecular “brick” (e.g., CH_2_) does not affect tunneling barrier height but rather only barrier length. A similar conclusion was reached by Hines at al. in a similar work analyzing ET rates vs barrier distance in CdSe QDs–(SH–[CH_2_]_*n*_–COOH)–TiO_2_ MO;^158^ and the same conclusion was derived by Anderson et al. when studying Re-based molecular dyes sensitizing a mesoporous SnO_2_ MO, specifically Re(CO)_3_Cl(dcbpy) [dcbpy = 4,4′-dicarboxy-2,2′-bipyridine] (ReCnA) with methylene units (CH_2_)_*n*_ (*n* = 1–5) inserted between the bipyridine rings and the carboxylate anchoring groups.^216^ The consistency of these results highlights the generality of the observation independently of the nature of the donor, either a molecular dye or an inorganic QD nanocrystal.^86^

Furthermore, it is worth highlighting here that Wang et al.^170^ report β figures (β_*n*_ = 0.94 ± 0.08 and β_*n*_ = 1.25 per methylene and phenylene group, respectively) that agree quantitatively with values also reported from conductance measurements through single molecules (measured in vacuum by scanning tunneling probes) and self-assembled monolayers.^217−221^ This agreement strongly supports the conclusions made in this work (together with the employed methodology) and indicates that conductance and ET rates through a molecular bridge are indeed closely correlated as theoretically predicted by Nitzan.^50^ This parallelism and link to the field of molecular electronics are worth highlighting as they pinpoint that molecular bridges between the QD donor and MO acceptor could, in theory, be engineered to have other functions beyond a resistor-like barrier potential. For example, a molecule displaying rectification between the donor and acceptor could be exploited for enhancing ET from the dot toward the oxide and inhibiting back ET from the oxide to the dot.^222−224^

**Figure 15:**
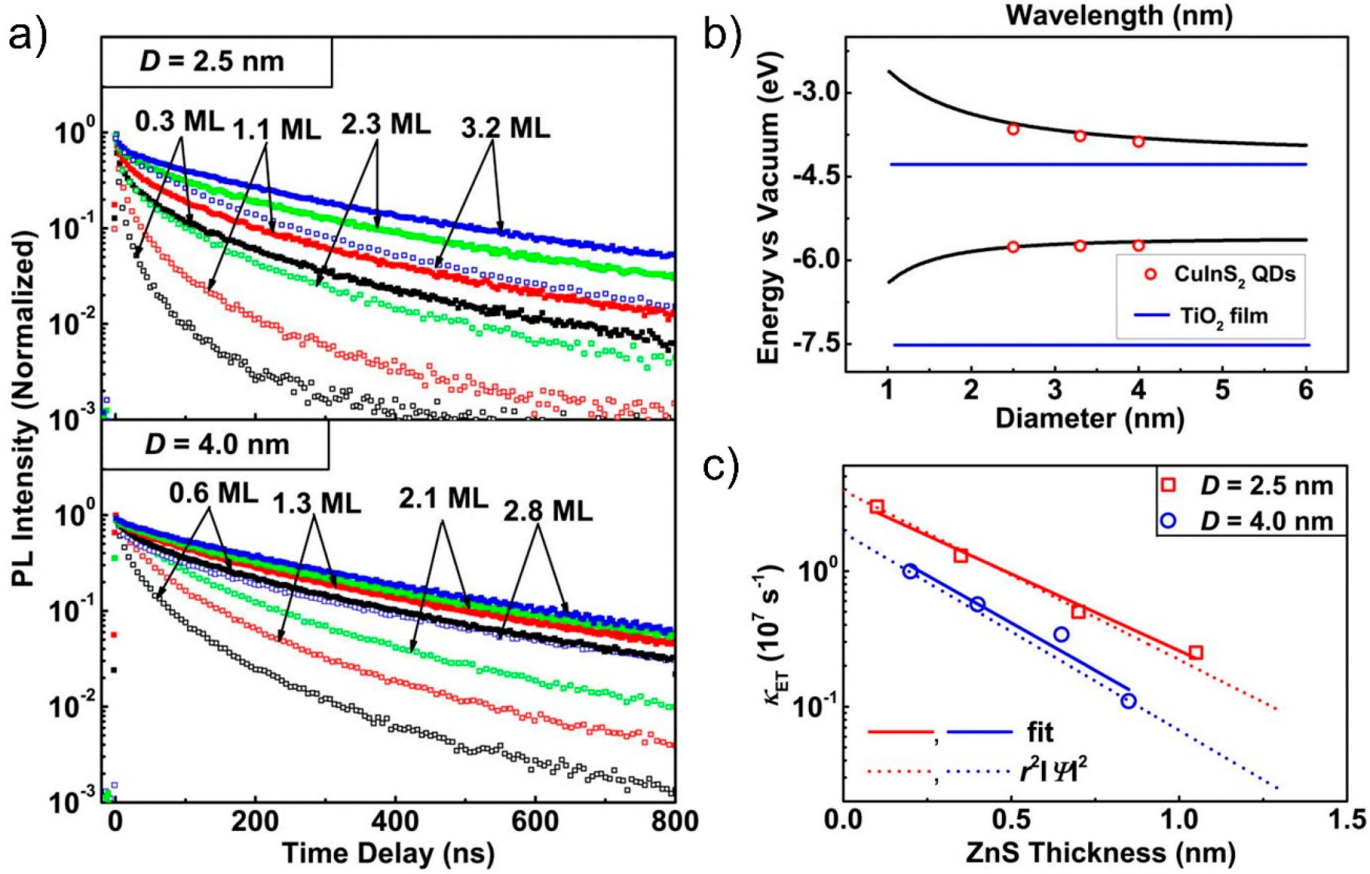
(a) PL decay curves of CuInS_2_/ZnS core–shell QDs with various core diameters and the ZnS shell thicknesses deposited on ZrO_2_ (solid dots) and TiO_2_ films (empty dots). (b) LUMO and HOMO levels of CuInS_2_ QDs shown by red circles as measured by cyclic voltammetry. Black lines represent LUMO and HOMO levels of the CuInS_2_ QDs. Blue lines represent the LUMO and HOMO levels of the TiO_2_ film measured by cyclic voltammetry and optical absorption. (c) Plots of ET rates of CuInS_2_/ZnS core QDs with core diameters of *D* = 2.5 nm (red squares) and *D* = 4.0 nm (blue circles) as a function of ZnS shell thickness. The solid line represents the fit of the ET rate. Calculated electron densities at the ZnS surface as a function of ZnS shell thickness are shown by dashed lines. The electron densities lines were normalized to the fastest measured ET rates for comparison. Reprinted from ref (211). Copyright 2013 with the permission of AIP Publishing.

As a final note, Sun et al.^211^ analyzed by TRPL shell-thickness-dependent photoinduced ET from CuInS_2_/ZnS quantum dots to TiO_2_ films (Figure 15). They demonstrated that the rate and efficiency of ET can be controlled by changing the core diameter and the shell thickness. They found that the ET rates decrease exponentially at decay constants of 1.1 and 1.4 nm^–1^ with increasing ZnS shell thickness for core diameters of 2.5 and 4.0 nm, respectively, in agreement with the electron tunneling model. Analogous results were obtained by Zhu et al. but in this case with a core–shell QD toward a molecular acceptor.^52^

### 6.3 Effect of Temperature on ET

Temperature is also a critical parameter to ascertain the nature of the mechanism determining ET at a given QD–MO interface (e.g., it can discriminate whether ET at the QD–MO interface occurs via coherent tunneling or hopping). However, to our knowledge, there are no experimental studies interrogating the interplay between ET rates and temperature at QD–MO interfaces beyond those analyzing hot electron transfer from the QD to the MO.^173,225,226^

The absence of experimental reports is likely due to the presumed complexity of varying the *T* of the system without affecting other parameters like energetics or coupling at the interface. For example, both QDs and MO band gaps manifest a *T* dependence, the strength of which is size-dependent for QDs.^227^ Also, hot carrier cooling within the QDs can be *T*-dependent for both processes, either for electrons relaxing in a continuum of states for high energies^228−230^ or between discrete levels close to the gap edge. This aspect was shown by Schaller and co-workers^93^ by studying TAS on a set of colloidal suspensions of PbSe and CdSe QDs of various sizes. The authors reported regions of *T* where the relaxation rate from the 1P toward the 1S state was thermally activated, with a clear QD size and material dependency linked to the process.

In the absence of experimental works, Tafen et al. approached the problem theoretically by simulating the explicit temperature dependence of ET from CdSe QDs to a TiO_2_ nanobelt.^201^ They combined time-domain density functional theory with nonadiabatic molecular dynamics to investigate the size and temperature dependence of the experimentally studied electron transfer and back electron transfer in the system. They show an electron injection rate with a strong dependence on the QD size, increasing for small QDs. Both transfer rates obtained from the simulations exhibit an Arrhenius-type temperature dependence with an activation energy of the order of millielectronvolts. Simulations suggest that temperature dependence of the back electron transfer rate can be successfully modeled using the Marcus equation (eq 1) through optimization of the electronic coupling and reorganization energy.

### 6.4 Effect of Density of Accepting States ET

**Figure 16:**
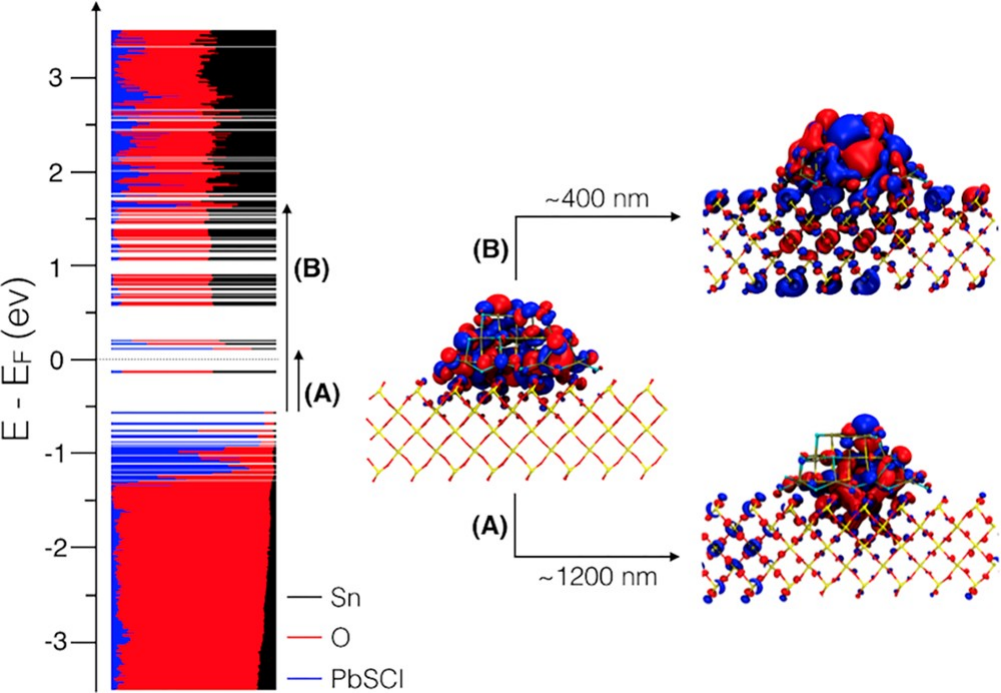
Enhanced wave function leakage for hot states in the QD depends on excess energy *E*_ex_ and/or enhanced DOS in boththe QD and the oxide. The colors indicate the moiety contributing to the orbital: PbS (blue), Sn (black), and oxygen (red). It is apparent that, for the CB region, the PbS states are evenly mixed with those of SnO_2_. This implies that the contribution to the coupling from wave function overlap is largely independent of *E*_ex_. This is further corroborated by the wave functions delocalized over both PbS and SnO_2_ for different excess energies. Reprinted from ref (173). Copyright 2018 American Chemical Society.

Marcus theory, as expressed in eq 1, has a neat dependence on the density of states ρ(*E*) of the acceptor. This question was also relevant in the dye sensitized ET community,^26^ where they qualitatively concluded that an increased DOS in TiO_2_ vs SnO_2_ could explain faster ET rates for the former independently of a reduced excess energy between the donor and acceptor states (this was phenomenologically linked with the CB of SnO_2_ being defined by s and p orbitals of the metals while the TiO_2_ CB is formed by empty d orbitals of Ti^4+^).^16,26^ For the QD–MO systems, while there are studies that have analyzed different oxides which are characterized by different densities of states, a rather qualitative comparison could be made at best.^154^ This is due to the fact that one should be very careful to compare such systems under different donor–acceptor energetics. A proper analysis of this parameter would require analyzing the ET toward oxides over the same, accurately measured, donor–acceptor excess energy Δ*G* and ideally with size-dependent studies. One theoretical analysis of the D–A coupling term in a given QD–MO interface as a function of excess energy (i.e., for an increasing DOS in the oxide) suggests indeed that increased ET rates as a function of excess energy (form hot states) can be primarily assigned to an increased density of states of the MO acceptor at higher excess energies (see Figure 16).^173^

### 6.5 Effect of Reorganization Energy ET

The reorganizational energy term λ is usually relevant when a D–B–A system is composed of, or is surrounded by, a large number of nuclear coordinates that need to be rearranged for the transfer of an electron to occur.^86,154,194,195^ The reorganizational energy is in principle contributed by outer and inner components; the first one is linked to the environment around the system (e.g., solvation), and the inner component refers to the degrees of freedom linked to D–B–A building blocks (e.g., QD phonons and/or molecular vibronic states) assisting eventually ET from the QD donor to the MO acceptor.

The outer sphere component toward the reorganization energy is expected to be smaller in QDs when compared with molecular dyes.^182^ Also, it is null in systems surrounded by vacuum when compared with those surrounded by a solvent.^225,231^ These qualitative aspects are in line with reports when comparing figures for dye and QD chromophore sensitizing MOs. Ai et al. reported λ values of ∼100 meV in dye-MO dry films,^231^ while Tvrdy and co-workers proposed values as small as 10 meV for their modeling of CdSe–MO films in vacuum.^154^ In line with this report, other authors have made quantitatively similar estimates (few tens of meV) about the reorganizational energy from fits of the many-states Marcus theory.^131,154,172,225^ In any case, one should take with caution any of these estimates coming from *K*_et_ fits to Δ*G* estimates, where Δ*G* was not properly addressed (inferred from isolated QD and MO workfunctions rather than those present at the sensitized interface; as already described in more detail in section 6.1).

**Figure 17:**
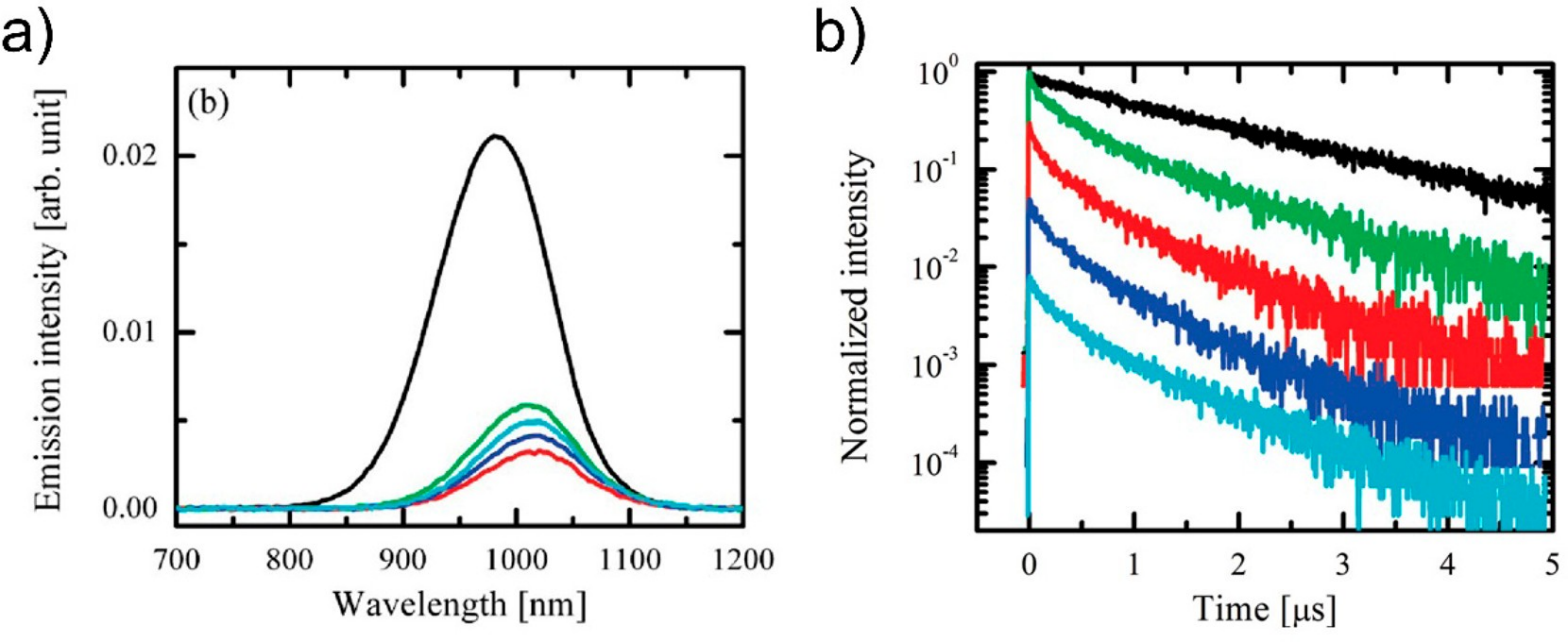
(a) Fluorescence spectra of 3.5 nm diameter PbS NCs in TCE (black) and PbS NC-MPA-TiO_2_ composites in TCE (green line), chloroform (red line), chlorobenzene (blue line), and dichloromethane (cyan line). Excitation wavelength is 780 nm. (b) Transient fluorescence traces of PbS NCs in TCE (black) and PbS NC-MPA-TiO_2_ composites in TCE (green), chloroform (red), chlorobenzene (blue), and dichloromethane (cyan). The bleach is consistent with ET. For clarity, the transients have been vertically displaced. Reprinted from ref (182). Copyright 2011 American chemical Society.

To our knowledge, a rational attempt to determine the impact of the solvent toward ET at a QD–MO interface was done by Hyun et al.^182^ (see Figure 17) that analyzed ET by TRPL from lead-salt QDs toward TiO_2_ (bridged by 3-MPA) under different solvents. The QD-MPA-TiO_2_ composites were dispersed in tetrachloroethylene (TCE), chloroform, chlorobenzene, and dichloromethane; the solvents were chosen to meet two conditions simultaneously: (1) PbS NCs coated with oleic acid should be well-dispersed in the solvents, and their optical properties should not change; and (2) the MPA-capped TiO_2_ nanoparticles should be well-dissolved in the same solvents. Unfortunately, many polar solvents with high static dielectric constants such as acetonitrile, dimethylformamide, and dimethyl sulfoxide could not be used due to the limited solubility of both the PbS NCs and MPA-capped TiO_2_ nanoparticles. In any case, the authors resolved that the fluorescence of the PbS NCs in TCE decays with a time constant of 1.7 μs (black line in Figure 17b). The fluorescence decays of the composite in other solvents are faster, but in different solvents they were almost the same. This insensitivity of ET vs solvent was attributed to the relatively small solvation term inferred for the reorganization energy (λ ∼ 30–100 meV); the weak dependence on the solvent dielectric constant was attributed to screening effects induced by the large size of the nanoparticles involved. These results are in line with the observed very modest solvatochromism effect seen in isolated QDs in solution by Leatherdale and Bawendi.^232^

As far as we know, little is known about the eventual impact to the reorganizational energy from the inner component, i.e., linked with phonons and molecular vibrational modes of molecular capping or bridge assisting ET. Substantial work has been done to try to understand how the relaxation dynamics of hot electrons are dissipated within the QDs. Some reports suggest coupling with collective modes of the QD or with more localized molecular vibrational states (these aspects are obviously largely dependent on sample nature and chemistry).^65,66^ Whether any of these photophysics might assist ET at QD–MO interfaces is yet to be unraveled.

## 7 Nonequilibrium (Nonthermalized) Electron Transfer at QD–MO Interfaces

The fundamental picture for interfacial dynamics drawn until now has been done by implicitly considering that electron transfer at the QD–MO interface takes place only once the photogenerated charge carriers populating the QD have reached a quasi-steady-state situation. Basically, when the electron and hole have dissipated, in the form of heat, all the excess energy above the HOMO–LUMO gap arises after the absorption of highly energetic photons. As such, electron transfer from the QD toward the oxide takes place from the QD-LUMO toward the MO conduction band, and the maximum efficiency that can be achieved at the interface is given by the Shockley and Queisser limit.^76^

Here will explore interfacial dynamics, from the perspective of kinetics, occurring under nonequilibrium, basically when hot non-thermalized electrons populating the QD can be transferred toward the MO, and/or when they trigger the generation of multiple excitons by impact ionization. Under these scenarios, QD–MO architectures can be employed to realize two third-generation photovoltaics concepts: hot carrier solar cells (by enabling hot electron transfer, HET)^109^ and carrier multiplication based solar cells (via multiple exciton generation, MEG).^45^ Both of these concepts, widely analyzed within the QD community, came from the initial expectations seeded by the unique photophysics induced by quantum confinement, most notably by the so-called “phonon bottleneck” effect. The phonon bottleneck effect refers to the expectation that hot carrier cooling should occur much slower in QDs than in their bulk counterparts.^91,92,233^ The idea at the base of this effect is that charge carrier excess energy relaxation from discrete energy levels, which are separated by multiple quanta of phonon energy, cannot sustain multi-phonon emission, i.e., the ultrafast deactivation pathway for hot electrons taking place in bulk materials. As such, the expectation was that stronger confinement (larger spacing between discrete energy levels) should be linked to reduced hot carrier cooling rates within the QDs and also that the lack of multiphonon emission processes could led to a most efficient impact ionization deactivation mechanism. Larger hot carrier lifetimes could facilitate their extraction (for hot carrier solar cells), and improved impact ionization could be exploited in carrier multiplication solar cells. While many works attempted to probe a phonon bottleneck effect in QDs, they generally resolved an opposite trend; i.e., the stronger the confinement and larger the separation of discrete energy levels, the faster the hot carrier relaxation was taking place.^93−100^

Several works rationalized these observations in terms of an Auger-like, coulombic interaction, where a hot electron populating discrete energy states within the dots gives the excess energy to its parent hole, which most generally is able to dissipate excess heat via phonon emission by a more dense distribution of energy levels (linked with heavier effective masses).^93−97,234^ Once the mechanism was cleared, a possible workaround to increase the lifetime of hot carriers can be to spatially separate e–h, e.g., in a core–shell geometry. This approach was used by Pandey and Guyot-Sionnest to demonstrate very large hot electron lifetimes in engineered QDs.^96^ On the other hand, the expectation of improved impact ionization yields induced by quantum confinement in QDs vs bulk materials was heavily scrutinized in the literature over the years.^93,104^ After the initial reports highlighting magnificent quantum yields for multiexciton generation in QDs,^235^ a strong debate surged in the research community regarding those claims.^101−104,236^ Later on, it was acknowledged that photocharging effects were masking true, more modest, MEG yields in QDs. At present, much work has been reported in this field, with systems engineered and showing optimized MEG yields but mostly on QD-based bulk-like solids, in which the generation of multiple excitons tends to quickly dissociate them into free carriers right after impact ionization takes place.^237,238^ After efficient MEG, charge transport can take place and extraction toward an external contact is conventional with the gain in photocurrent associated with the MEG process. On the contrary, at a QD sensitized MO interface, multiple excitons, eventually generated by MEG, will populate an isolated QD, generating localized bound states at the interface. These multiple excitons will quickly recombine via Auger processes, and then, any attempt to collect biexcitons at the interface will require ultrafast extraction toward the MO. To summarize, the challenge is the need for ultrafast exciton dissociation at QD–MO interfaces to compete with ultrafast carrier cooling of hot carriers and exciton–exciton annihilation. In the following, we will highlight key works realized for both approaches.

### 7.1 Hot Electron Transfer from QDs to MO

In order to achieve an efficient hot electron transfer (HET) at any donor–acceptor interface, it is mandatory to either increase the hot electron lifetime in the donor or to enhance the ET speed toward the oxide. Improved lifetimes of the hot non-thermalized electrons in QDs can be tackled by, e.g., the spatial e–h separation achievable in core–shell QDs.^96^ On the other hand, according to the many-states Marcus theory (see eq 1), boosting ET toward the oxide can be achieved by enhancing donor–acceptor coupling strength. This aspect can be attempted by employing short linkers between the QD and MO, by using oxides with larger DOS for a given excess energy, or by employing QDs in the strong quantum confinement regime (i.e., with an improved wave function leakage outside the dot). These reasons led many authors to work primarily on lead salt based QDs sensitizing TiO_2_ electrodes to prove the feasibility of the process. It is worth noting here that, to achieve any efficiency gain linked to HET, one should not dissipate that gain in the MO electrode as heat (i.e., the collected hot electron should not loss its excess energy in the contact). From all the works discussed below, only one example might offer the proper interfacial energetics.^225,226^

**Figure 18:**
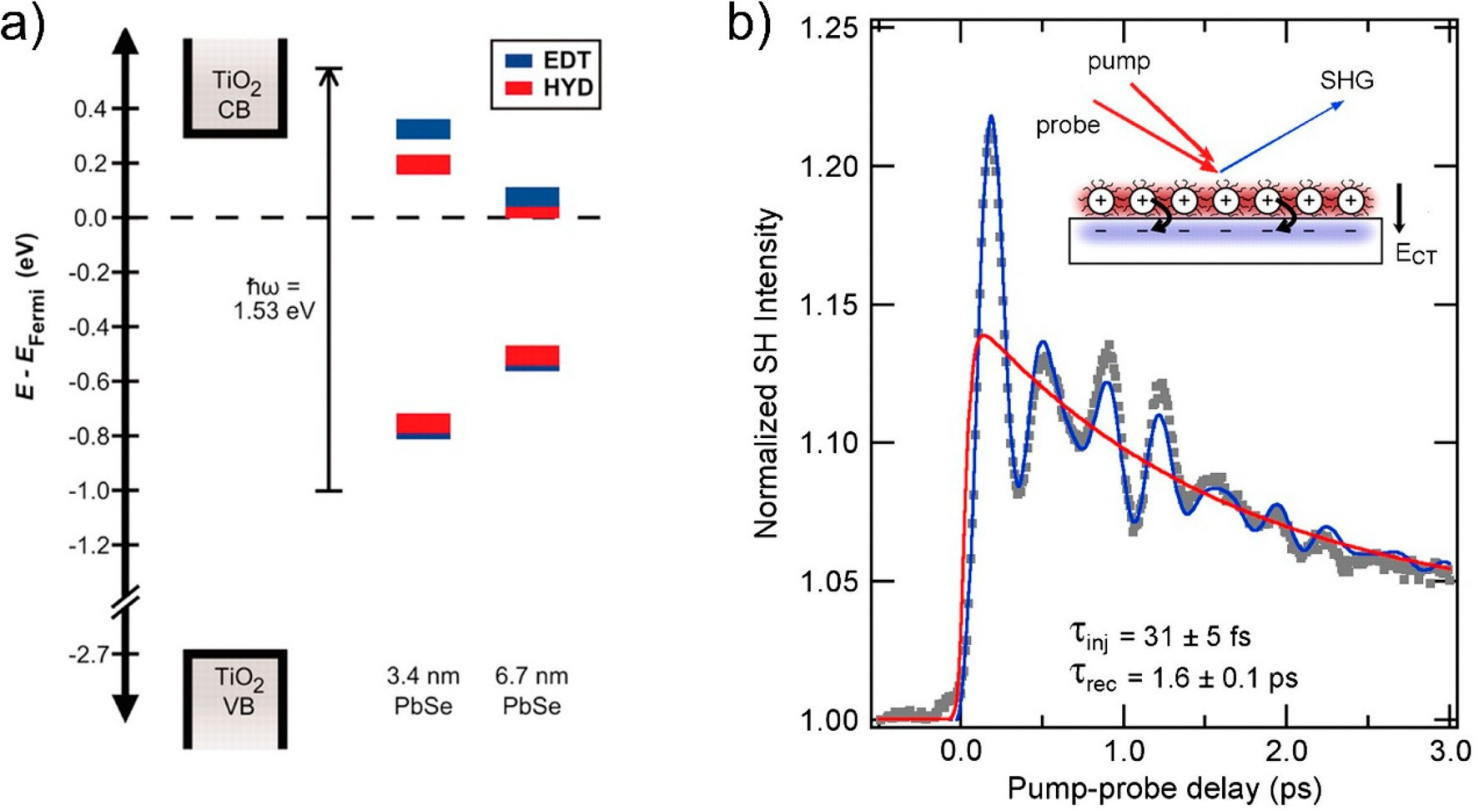
(a) Alignment of highest occupied and lowest unoccupied quantum dot energy levels relative to the TiO_2_ conduction band edge after chemical treatment of the nanocrystal surface. Alignment is determined by ultraviolet photoelectron and near-infrared absorption spectroscopies and indicates that electron transfer from the lowest excited state of the quantum dot is not energetically possible. The vertical arrow depicts symmetric photoexcitation of the PbSe quantum dots with 810 nm light. VB, valence band; CB, conduction band; EDT, 1,2-ethanedithiol; HYD, hydrazine. From ref (226). Copyright 2010, reprinted with permission from AAAS. (b) Time-resolved SHG (dots) of the TiO_2_ surface coated with 1.5 monolayers of EDT-treated 6.7 nm PbSe nanocrystals. The sample temperature was 12 K. Both pump and probe were 50 fs pulses of 810 nm light. The intensity of the reflected second harmonic light at 405 nm was recorded as a function of time delay between the pump and probe pulses. The blue curve shows a least-squares fit incorporating electron injection and recombination (red) and three coherent phonon modes. From ref (226). Copyright 2010, reprinted with permission from AAAS.

The first report about HET from a QD nanocrystal to a MO dates to 2010, when Tisdale et al. published their observations using second harmonic generation (SHG) on a system consisting of one (or two) monolayer of PbSe QD sensitizing an atomically flat single-crystalline (110) rutile TiO_2_ surface.^225,226^ The films were also chemically treated with either hydrazine or 1,2-ethanedithiol (EDT) with the aim to respectively remove or substitute the more insulating oleic acid capping present on the QD surface and improve the coupling between the nanoparticles and the MO. The authors were able to follow a build up represented by HET and the decay of the SHG signal given by either recombination, back ET, or diffusion of the transferred electron into the bulk of TiO_2_. Analysis of the data (see Figure 18b) revealed an electron injection time constant of 31 ± 5 fs and a combined recombination and diffusion rate of about 1.6 ± 0.1 ps. The observed HET process was more prominent at low temperature (80 K), as expected when decreasing carrier cooling rates that directly compete with electron injection.^93^ As stated before, this system offers the proper interfacial energetics required by the HCSC concept. However, the observation is made on a flat single-crystal facet. Hence, if exploited in a real device, the photocurrent generated is expected to be almost null due to the small sensitizer loading.

Yang et al. presented a study on ET resolved by TAS between PbS QDs sensitizing nanocrystalline, rather than bulk, TiO_2_ films.^157^ They estimated a 6.4 fs electron injection characteristic time from the 1S electron level of PbS QDs to TiO_2_ nanocrystalline thin films. This rate was estimated from the broadening of the absorption band of the QDs that was assigned to the coupling with the MO, as predicted by the Newns–Anderson model for chemisorption. Although the authors do not observe a signature that can be imputed without any reasonable doubt only to HET, due to the limited time resolution of the experimental apparatus, the fast femtosecond (<150 fs) electron injection rate that they report is consistent with the one reported by Tisdale et al. for hot electron injection from PbSe to a rutile (110) TiO_2_ surface, suggesting the feasibility of hot electron extraction from photoexcited lead salt QDs toward TiO_2_.^226^

Cánovas et al. also devoted some attention to the topic of HET, investigated by the means of TRTS. They studied whether HET was taking place from the 1P_e_ states of colloidal 3 nm PbSe QDs molecularly linked by MPA to mesoporous SnO_2_ and TiO_2_ sensitized films.^191^ The authors purposely photo-oxidized their samples during data collection in an attempt to discriminate between HET and any parasitic signals obscuring the process (often affecting early pump–probe dynamics). From their data, the authors concluded that a HET yield from the 1Pe state of the 3 nm PbSe QDs toward the TiO_2_ CB reached about 80%, while the efficiency was reported to be almost null (lower than 10%) in the case of the SnO_2_. These findings were rationalized by the distinct QD-oxide coupling strength for different systems.

**Figure 19:**
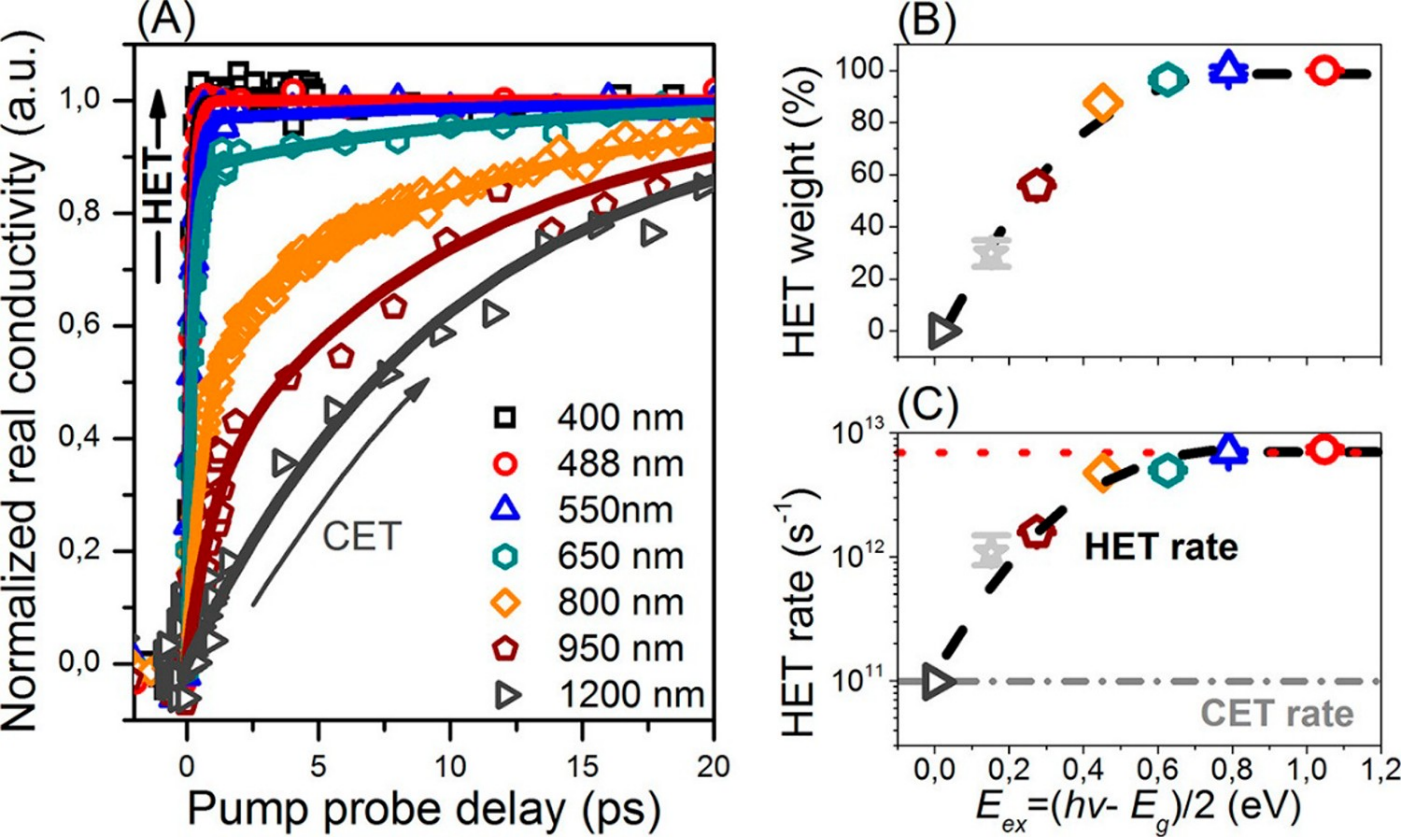
Photon-energy-dependent hot electron transfer (HET). (A) Excitation wavelength-dependent ET dynamics (from 400 to 1200 nm) for the sample C3 (PbS QDs with ∼2.7 nm diameter). The solid lines are biexponential fits based on hot and cold electron transfer (HET, on sub-picosecond time scales, and CET, on an ∼10 ps time scale; see arrows). (B) Weight of the fast HET component in the dynamics shown in panel (A) vs hot electron excess energy in the QDs. (C) HET rates vs the excess energies of hot electrons. The CET rate was found to be independent of excess energy and fixed to 10.2 ps (gray dotted-dash line). In panels (B) and (C), the dashed black line is to guide the eye; the red dotted line represents the time resolution of our setup. Reprinted rom ref (173). Copyright 2018 American Chemical Society.

Wang et al. tried boosting HET collection by exploring the case of PbS QDs grown in situ by SILAR onto a mesoporous SnO_2_ matrix.^173^ A merit of this work was that, by employing an increasing photon energy pump above the HOMO–LUMO gap, they observed a transition in the ET dynamics from a single to double exponential process, which they could unambiguously assign to cold electron transfer (CET) and HET, respectively (Figure 19A). With the support of TRTS and DFT simulations, they demonstrated that the HET rate and HET collection efficiency were substantially enhanced when the hot electron possesses higher excess energy from the QD-LUMO (Figure 19B,C). They rationalized that this phenomenon is due to an increased density of acceptor states at higher energies (see Figure 16). When photon energies were in excess of ∼0.5 eV with respect to the HOMO–LUMO gap of the QD, the authors studied a setup limited sub-150 fs HET process at room temperature with a unity quantum yield for the studied system. Additionally, they observed an increasing HET efficiency at lower temperatures consistent with a reduced hot carrier cooling rate within the QD.

### 7.2 Carrier Multiplication: Multiple Exciton Generation (MEG)

MEG requires that the excess energy of a photogenerated electron has to be given to a second electron–hole pair, rather than be transferred to the conjugated hole.^45,104^ After the multiple exciton formation in the QD, the extraction should be faster than exciton–exciton annihilation.^188^ Analogously to the case of HET, one could attempt achieving high multiexciton collection efficiency at a sensitized interface in two ways, by reducing exciton–exciton annihilation lifetimes or by boosting the coupling between multiexcitonic states in the donor and the continuum of states in the MO conduction band. There are a few examples of multiple exciton collection in a working photovoltaic device. For instance, Sambur and co-workers reported the use of a photoelectrochemical system composed of colloidal PbS dots sensitizing via MPA to TiO_2_ anatase single crystals to demonstrate the collection of photocurrents with quantum yields greater than one electron per photon absorbed.^42^ Though remarkably proving the possibility to have internal quantum yield above unity, the authors raise concerns about the effective device improvement because of the onset of MEG is at nearly 3 times the QD band gap.

**Figure 20:**
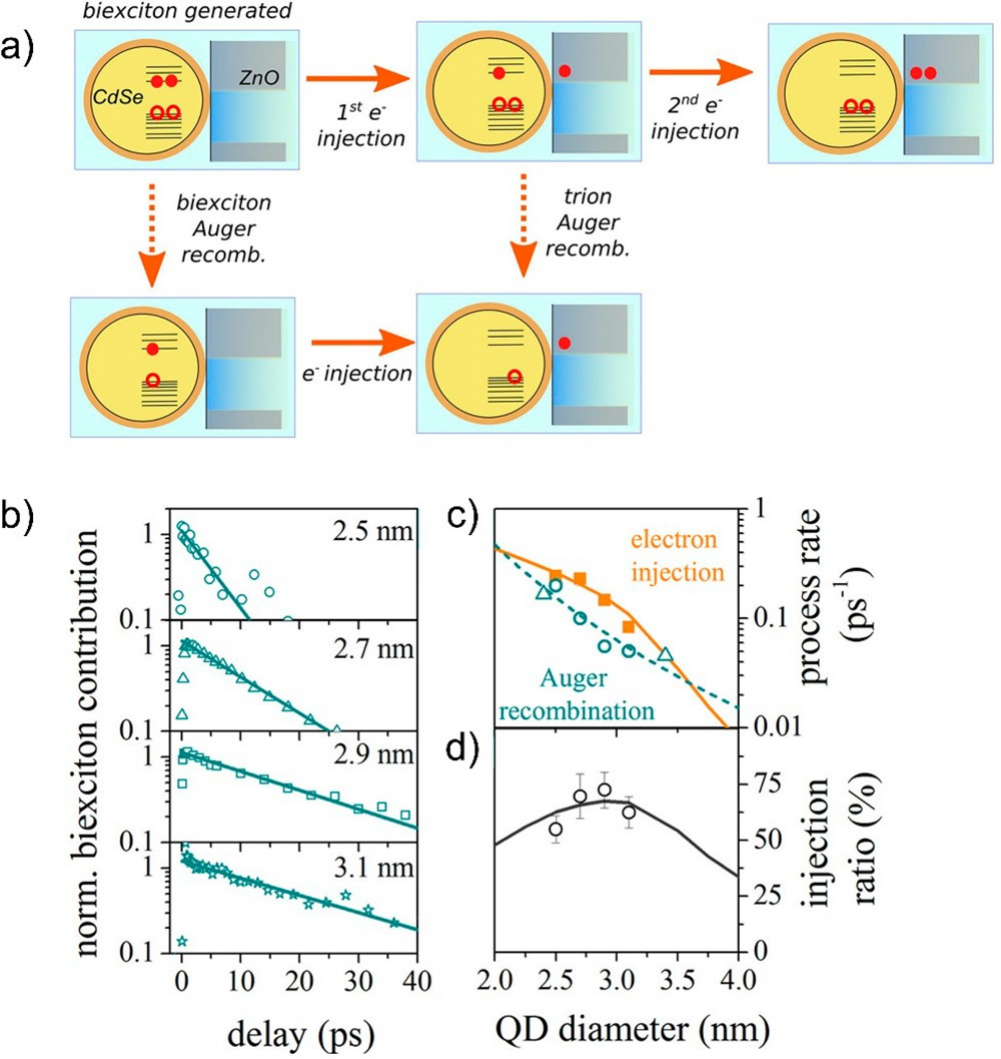
(a) Electron injection and Auger recombination processes present in a QDs attached to ZnO. (b) Normalized biexciton Auger contribution to TA kinetics for various QD diameters (open symbols) fitted by single-exponential decay (solid lines). (c) Electron injection rate dependence on QD size (orange squares) fitted by the Marcus theory prediction (solid orange line); Auger biexciton recombination rates from the fit in panel (b) (dark cyan circles). Dark cyan triangles show fitting by *D*^–*p*^ (*p* = 4.9, dark cyan line). (d) First electron injection efficiency from QDs populated with biexcitons for various QD sizes calculated from experimental data (open circles) and fits presented in panel (c) (line). Adapted rom ref (239). Copyright 2012 American Chemical Society.

To our knowledge, only two papers have analyzed carrier dynamics linked to multiple exciton collection in QD–MO interfaces.^174,239^ However, in both cases, they achieved a sizable biexciton population via two-photon absorption processes in the QDs, i.e., by employing extremely high photon fluxes rather than by MEG processes under UV illumination. Žídek et al. performed an extensive study about multiple exciton collection in a colloidal CdSe on ZnO.^239^ The authors proposed a model to describe the kinetic processes involved in multiexciton collection, which are summarized in Figure 20a. The biexciton is transferred to the MO in two different steps, one electron at a time, while competing with both biexciton Auger lifetime and the positive trion recombination rate. By measuring dynamics with TAS, the authors concluded from modeling that a biexciton harvesting efficiency from 30% to 70% was achieved with QDs sizes ranging between 2 and 4 nm. These variations were linked to a trade-off between size-dependent Auger recombination and Marcus-like driving force ET toward the oxide electrode (Figure 20b–d).

**Figure 21:**
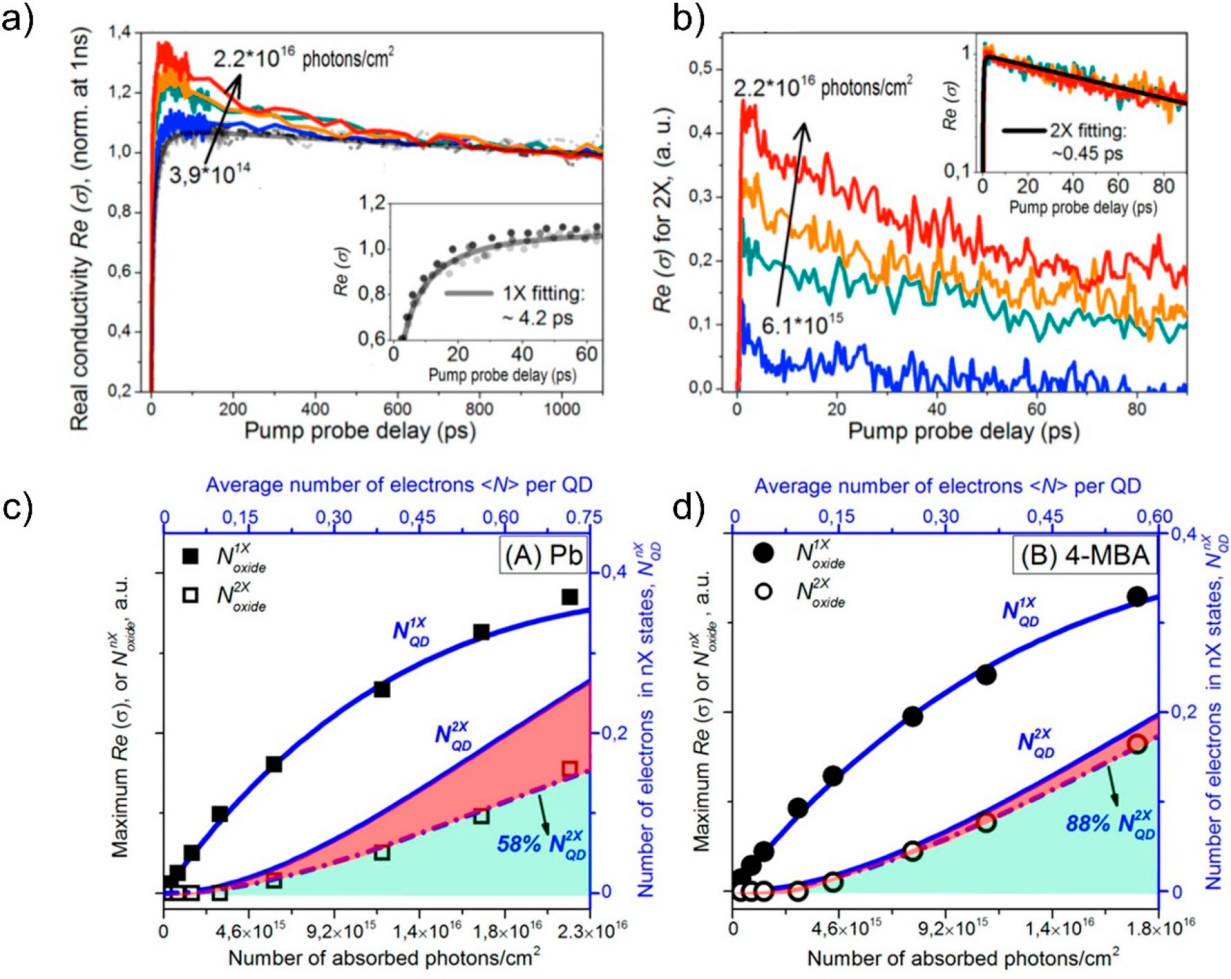
(Top) ET dynamics for PbS QDs sensitizing a SnO_2_ mesoporous film as a function of photon flux with *hν*/*Eg* < 2 photon excitation. (a) Time-resolved photoconductivity data normalized to values at 1 ns for several 800 nm pump excitation fluences. The black line represents the best fit to single-exciton (1×) dynamics, and the inset shows single-exciton early time dynamics. (b) Inferred biexciton (2×) dynamics. The inset shows dynamics normalized to the peak signal, and the black line represents the model calculation described in the reference. (Bottom) Multiexciton collection efficiency for PbS QDs sensitizing a SnO_2_ mesoporous film as a function of photon flux. (c) Poisson statistics of the number of electrons in *N*_QD_ 1× and *N*_QD_ 2× states populating QDs after excitation. The difference between biexcitons photogenerated in the QDs and transferred into the oxide represents losses in the QDs via Auger recombination (red area). The highlighted green area shows the additional electrons transferred by photogenerated biexcitons in the oxide electrode. (d) Same plot as in panel (c) but for a sample where QDs sensitizing SnO_2_ are passivated by 4-mercaptobenzoic acid (4-MBA), clearly leading to enhancement of the biexciton collection efficiency. Adapted with permission from ref (174). Copyright 2017 American Chemical Society.

Wang et al. did a similar study by demonstrating the collection of multiple excitons from a system consisting of PbS QD grown in situ by SILAR onto a mesoporous SnO_2_ matrix (Figure 21).^174^ As stated before and analogously to the work discussed above, the authors did not achieve multiple excitons in the QDs via MEG (with photon energies exceeding at least twice the QD gap), but rather focused on the biexciton collection at the QD–MO interface following the sequential two-photon absorption near the QD HOMO-LUMO gap. With TRTS, they were able to quantify precisely the amount of electrons reaching the MO for a given photon flux by spectrally resolving the fingerprint of ET for QDs populated by a single exciton or biexciton (Figure 21a,b). Notably, by ligand engineering of the QD capping, they demonstrated a boost in biexciton collection efficiency (Figure 21c,d). They rationalized the finding to partial localization of holes in the molecular shell, a factor that enabled a reduction of Auger recombination in the dots and then extended biexciton lifetimes.

## 8 Summary and Outlook

What emerges from this Review is that our fundamental understanding of the mechanisms of charge transfer at QD–MO interfaces is far from being complete. Several hints, such as major discrepancies between electron transfer rates in apparently similar systems, point to the fact that our current knowledge is rather qualitative. We believe that to a large extent these discrepancies are likely associated with several methodology pitfalls described in this review, affecting all methods and experimental approaches in both general and singular ways. Another critical aspect that can be linked with the disparity of results coming from apparently similar systems is an improper definition of the samples, e.g., providing ET rates without properly characterizing and univocally assigning QD–MO interfacial energetics. Also, the role of defects on kinetics are normally not carefully considered in the conducted experiments; this is a critical aspect as defects are ubiquitous in the samples and can be induced after functionalization of the oxides or even during experiments via photodegradation. Critically, the kinetic fingerprints of ET form the QD to the MO and trapping are the same for most experimental methods.

The difficulties of the interpretation of most of the experimental data leads often to the assumption that two systems from two groups cannot be compared quantitatively. We put the reader in front of this issue. We believe that a proper description of the analyzed systems, together with careful design of experimental methodologies, will set the path to solve the impasse and to further the development of fundamentals and applications. For example, carefully corroborating that the samples are measured in the linear regime and employing when possible tailored energies ensuring selectivity for both pump and probe pulses will largely remove spurious signals arising in the experiments. On the other hand, issues like sample degradation or preparation history largely affect dynamics, and we showed how this is partially neglected in the literature. Several authors showed how the promotion of defects under controlled conditions can be turned into a useful tool to distinguish the various physical processes that happen at the QD–MO interface. In terms of promoting applications and establishing neat correlations between interfacial dynamics and device efficiency, more studies with complete in operando systems will be valuable.

The research in the field of ET at QD–MO interfaces extended naturally from the previous work on dye-sensitized systems. It is likely that both approaches are governed by identical theoretical backgrounds, with some differences deriving from the specificity of molecular sensitizers against inorganic QDs. By our judgment, anecdotal connections to the Marcus theory have been made in electron transfer studies in sensitized systems and specifically at QD–MO interfaces, normally with not well-defined systems and over quite limited ranges for the relevant parameters, insufficient to validate the theory. However, among the scrutinized parameters, the observation of the theoretically expected exponential dependence between ET rates and the distance imposed by the molecular bridge in QD–MO systems stands. Note that this dependence has been independently verified in dye-oxide, QD-oxide, single molecules, and self-assembled monolayers from different methods and experimental approaches. As such, we propose here that measuring this dependence on QD-(SH–[CH_2_]_*n*_–COOH)-MO systems should serve as a good protocol for validating any methodological approach made in any lab attempting the characterization of QD–MO interfaces (as is currently done in the field of single molecular electronics). The parallelism of interfacial kinetic studies and the field of single molecular conductance is worth highlighting, as it pinpoints that molecular bridges between a QD donor and MO acceptor can be engineered to have other functions beyond a resistor-like barrier potential; e.g., a molecule displaying rectification between the donor and acceptor could be exploited for enhancing ET from the dot toward the oxide and inhibiting back ET from the oxide to the dot.

At last, we have surveyed the most common routes analyzed in QD sensitized MOs to bypass the current limitations imposed by the Schockley–Queissier limit. Several reports provide mounting evidence about the feasibility of hot electron transfer and multiple exciton collection at QD–MO interfaces. However, in both cases, experiments have been performed with model systems that differ from the needs required by their respective theories; e.g., biexciton collection was achieved by artificially generating biexcitons by two-photon absorption in the QDs rather than by MEG, and HET was commonly reported for systems where energy gain induced by HET is lost as heat in the MO electrode (i.e., QD–MO interfacial energetics should be such that a hot electron is transferred to a selective contact without energy loss). Much work is still needed to validate the potential energy gains in better defined experiments. As an outlook, beyond MEG and HET approaches, one can envision other possibilities for third-generation concepts based on QD–MO architectures; e.g., nanocrystals showing up conversion can be employed in sensitized interfaces,^240^ or tandem QD structures (mimicking the Z-scheme in photosynthesis) functionalizing a mesoporous MO could enable novel routes toward device efficiencies beyond the Shockley–Queisser limit.
